# Tetrazine-Containing Amino Acid for Peptide Modification and Live Cell Labeling

**DOI:** 10.1371/journal.pone.0141918

**Published:** 2015-11-04

**Authors:** Zhongqiu Ni, Lanxia Zhou, Xu Li, Jing Zhang, Shouliang Dong

**Affiliations:** 1 Institute of Biochemistry and Molecular Biology, School of Life Sciences, Lanzhou University, Lanzhou, China; 2 The Core Laboratory of the First Affiliated Hospital, Lanzhou University, Lanzhou, China; 3 Key Laboratory for Gastrointestinal Diseases of Gansu Province, Lanzhou University, Lanzhou, China; 4 Key Laboratory of Preclinical Study for New Drugs of Gansu Province, Lanzhou University, Lanzhou, China; University of Helsinki, FINLAND

## Abstract

A novel amino acid derivative 3-(4-(1, 2, 4, 5-tetrazine-3-yl) phenyl)-2-aminopropanoic acid was synthesized in this study. The compound possessed better water-solubility and was synthesized more easily compared with the well-known and commercially available 3-(p-benzylamino)-1, 2, 4, 5-tetrazine. Tetrazine-containing amino acid showed excellent stability in biological media and might be used for cancer cell labeling. Moreover, the compound remained relatively stable in 50% TFA/DCM with little decomposition after prolonged exposure at room temperature. The compound could be utilized as phenylalanine or tyrosine analogue in peptide modification, and the tetrazine-containing peptide demonstrated more significant biological activity than that of the parent peptide. The combination of tetrazine group and amino acid offered broad development prospects of the bioorthogonal labeling and peptide synthesis.

## Introduction

Proteins/peptides performed various functions in living organisms, and it has been established that high-efficient and selective labeling of proteins/peptides were of tremendous importance to biomedical and biotechnological applications [1, 2]. However, the study of biomolecules in the physiological environments was a challenge because of the great complexity of biological systems [3]. Fortunately, bioorthogonal reaction has been developed to overcome these obstacles [4–7] and peptide modification has become an attractive area [8]. The most popular technique in the peptide modification was based on the introduction of genetically encoded non-canonical amino acids into proteins via bioorthogonal tRNA/tRNA-synthetase pairs [9]. The crucial method was the incorporation of amino acids with unique side-chain functionalities, which can be selectively connected by bioorthogonal chemical reactions [10]. To date, one of the successful applications in peptide modification was the using of the non-canonical amino acids [11–14]. The peptide modification in the future would be limited by the synthetic ability of the useful non-canonical amino acids [15].

Currently, it was reported that tetrazines reacted with strained alkenes rapidly and specifically via inverse electron demand Diels-Alder (IED-DA) cycloaddition reactions and generated stable adducts [4]. The rate constants were orders of magnitude faster than other established bioorthogonal reactions [16, 17]. Thus gene-encoding non-canonical amino acid that based on IED-DA cycloaddition reactions was considered as an attractive strategy to achieve the rapid and site-specific labeling of proteins [18]. Some dienophiles, such as trans-cyclooctene and norbornene, had stable functionality and were also easy to synthesize [5, 19, 20]. To date, numerous dienophiles-containing amino acids have been synthesized and incorporated into proteins in E. coli and mammalian cells through suppression of the Amber stop codon [21–23]. The stability of tetrazines can be dramatically increased with the substitution of aromatic groups, which increased the size and steric hindrance of the reactive moiety significantly [24, 25]. However, only a few tetrazine-containing amino acids have been applied to the biological environments for bioorthogonal labeling [9]. Recent work from Ryan Mehl has developed a tetrazine-containing amino acid and site-specifically encoded this unique amino acid in a protein [9]. Most of the literatures about the stabilities of tetrazine derivatives were measured in phosphate buffer saline (PBS) or 100% fetal bovine serum. Whereas there was no detailed investigation report on whether tetrazine can exist in 20% piperidine/DMF or 50% TFA/DCM stably with the deprotection of fluorenylmethyloxycarbonyl (Fmoc) or tert-butoxycarbonyl (Boc) group. Stepwise conjugation of tetrazine-containing amino acid through peptide synthesis was a formidable challenge.

Many synthetic non-canonical amino acids were connected by a ligand or a linker between these functional tags and the amino acid group, which was relatively bulky and difficult to be moved to multiple locations within peptides [22, 26]. To surmount these limitations, we set out to develop some simple amino acid derivatives, which can be connected directly between functional groups and amino acid without ligand, such as methylene group or amide group.

## Materials and Methods

All animal procedures and experimental protocols received approval of the Committee for Medical Ethics of Lanzhou University and the guidelines from International Association. The mice were supplied with food and water ad libitum. All efforts were made to minimize the number and any suffering of the animals used in the experiments.

### Chemicals, cells and animals

All chemicals were purchased from Sigma Aldrich unless noted. All reagents and solvents were reagent grade or were purified by standard methods before use.

A549 lung cancer cells were purchased from the Cell Bank of the Chinese Academy of Sciences (Shanghai, China).

Male Kunming mice (20 ± 2.0 g) from Animal Center of Lanzhou University (Lanzhou, China) were housed at the room temperature of 22 ± 1°C with a 12-h light-dark cycle and 50–60% relative humidity.

### Methods and apparatus

Column chromatography was carried out on flash silica gel (Sorbent 230–400 mesh). TLC analysis was conducted on silica gel plates (Sorbent Silica G UV254). Reverse phase high performance liquid chromatography (RP-HPLC) was carried out with Waters 600. Analytical HPLC was performed on a Sunfire^TM^ C18 Column (5 μm, 4.6 × 150 mm) at a flow rate of 1 ml/min measured by Waters 2996 Diode Array Detector. Preparative HPLC was performed with a COSMOSIL 5C18-AR-300 Column (5 μm, 20 × 250 mm) at a flow rate of 8 ml/min measured by Waters 2996 Diode Array Detector. For all HPLC, solvent A (water with 0.1% TFA) and solvent B (acetonitrile with 0.1% TFA) were utilized. All UV/vis spectra and kinetics experiments were recorded on an Agilent 8453 UV/vis Spectrophotometer. Pseudo first order rate constants from all kinetics experiments were calculated using the Agilent UV/vis Chemstation software package Rev. A.10.01. Fluorescence measurements were obtained using a Varian Cary Eclipse Fluorescence Spectrophotometer. ^1^H (300 MHz) and ^13^C nuclear magnetic resonance (NMR) (100 MHz) spectra were collected on a Bruker Spectrometer at the ambient temperature in DMSO. High-resolution electrospray ionization (ESI) mass spectra were obtained on a Bruker Daltonics Esquire 6000 with ESQ6K Operator. BL-420E+, Taimeng Technology Corporation of Chengdu, China.

## Results and Discussions

### Synthesis and enantiomeric purity analysis of tetrazine-containing amino acid 2 (S1 File)

**Fig 1:**
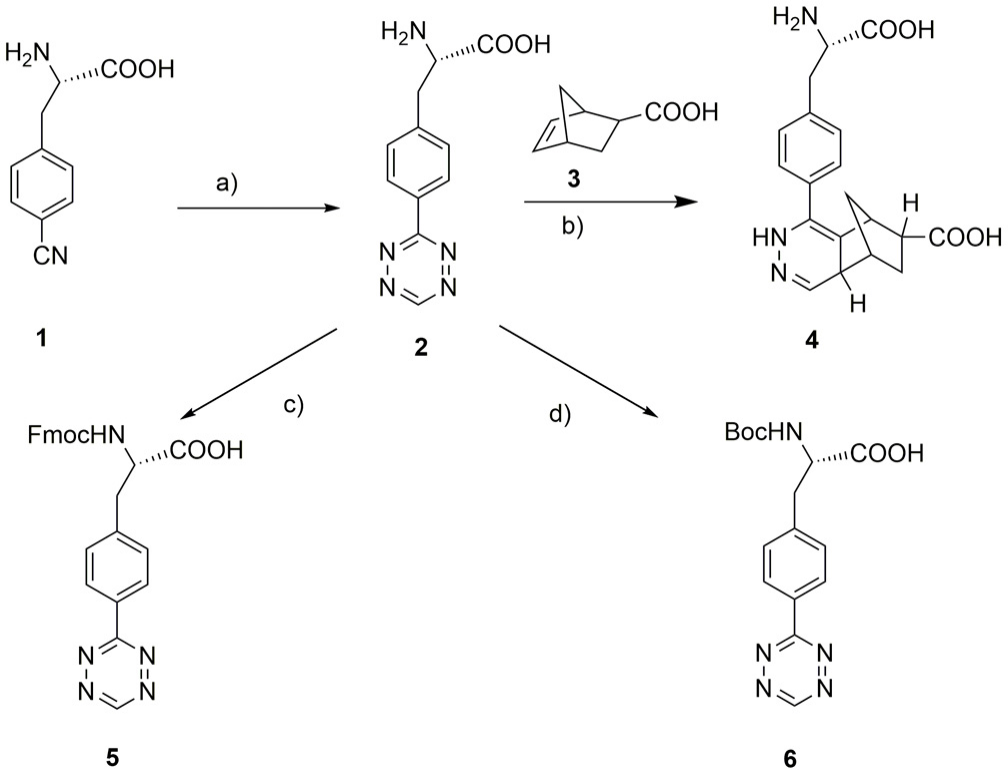
Synthetic route of tetrazine-containing amino acid (S)-3-(4-(1, 2, 4, 5-tetrazin-3-yl) phenyl)-2-aminopropanoic acid 2 and protected tetrazine-containing amino acid 5 and 6, and the IED-DA reaction of 2 with 5-norbornene-2-carboxylic acid 3 give 4. a) (i) Formamidine acetate (4 eq), N_2_H_4_ (40 eq), S (1 eq), r.t, 22 h; (ii) CH_3_COOH, NaNO_2_ (5 eq), 0°C, 30 min, 64.9%. b) Phosphate buffered solution (PBS), r.t, 30 min. c) Fmoc-OSu (1.5 eq), 1: 1 Dioxane/H_2_O (V/V), NaHCO_3_ (2.5 eq), 0°C, overnight, 64.5%. d) Boc_2_O (2 eq), 1: 1 Dioxane/H_2_O (V/V), NaHCO_3_ (2.5 eq), 0°C, 8h, 72.3%.

In this study, we connected tetrazine group to the benzene ring of phenylalanine directly and synthesized a tetrazine-containing amino acid (S)-3-(4-(1, 2, 4, 5-tetrazin-3-yl) phenyl)-2-aminopropanoic acid **2**, which featured two separate components: a phenylalanine and a tetrazine. The compound was synthesized from the commercially available 4-cyano-L-phenylalanine **1** with formamidine acetate and anhydrous hydrazine in the presence of elemental sulfur. The initial products dihydrotetrazine derivatives were oxidized to the tetrazines by treating with sodium nitrite in acetic acid (Fig 1) [27]. The pure product tetrazine-containing amino acid was generated as purple powder after the ethanol washing and filtration treatment. The purity was more than 95% in analytical RP-HPLC (**Fig A2 in S1 File**) and can be measured directly by NMR (**Fig B in S1 File**). Accordingly, the synthetic part of tetrazine-containing amino acid was much simpler than the well-known and commercially available 3-(p-benzylamino)-1, 2, 4, 5-tetrazine. The method may also be applicable for the synthesis of other tetrazine-containing amino acids.

The synthesis of tetrazine amino acid was very simple. However, because of the hydrazine, a kind of strong reducing agent, the reaction of cyano group released a lot of heat [28, 29]. In the whole reaction process, what we most concerned about was whether the configuration of L-amino acid changed. Marfey’s reagent 1-fluoro-2, 4-dinitrophenyl-5-L-alanine amide (FDAA) was selected for chiral analysis to test any configuration changes of the product [30]. The results of the enantiomeric purity confirmed that there was no configuration change of L-amino acid in the experiment. Further work would to be studied to understand the stability of **2** in IED-DA reaction, a prerequisite of in vivo applications.

### Stability and kinetic experiments of tetrazine-containing amino acid 2 (S2 File)

Unstable 1, 2, 4, 5-tetrazine could be stabilized with the substitution of aromatic rings [24, 25, 27], which might contribute to the application in bioconjugation. The compound **2** showed excellent stability in PBS with little decomposition after prolonged exposure at room temperature (**Fig G in S2 File**). To explore the feasibility of the tetrazine-dienophile reaction for biological labeling further, norbornene derivative was considered as a model dienophile substrate. Norbornene offered an excellent balance between facile strain-promoted reactivity with tetrazines and overall chemical stability [7]. And it was also easily available, stable and comparatively small in size compared with trans-cyclooctene [19]. Following with the exponential decay of the tetrazine absorbance at 523 nm upon reaction with a 10 fold excess of 5-norbornene-2-carboxylic acid **3** in water, we determined the reaction rate constant of norbornene as (1.339 ± 0.003) _M_^-1^·_S_^-1^ (**Fig H in S2 File**). The obtained reaction rate was similar to the reaction rate of 5-norbornene-2-carboxylic acid with dipyridyltetrazine (k_2_ = 1.3 ± 0.02 _M_^-1^·_S_^-1^) [19], suggesting its suitability in rapid biological labeling.

### Tetrazine-containing amino acid 2 in the lung cancer cell labeling (S3 File)

**Fig 2:**
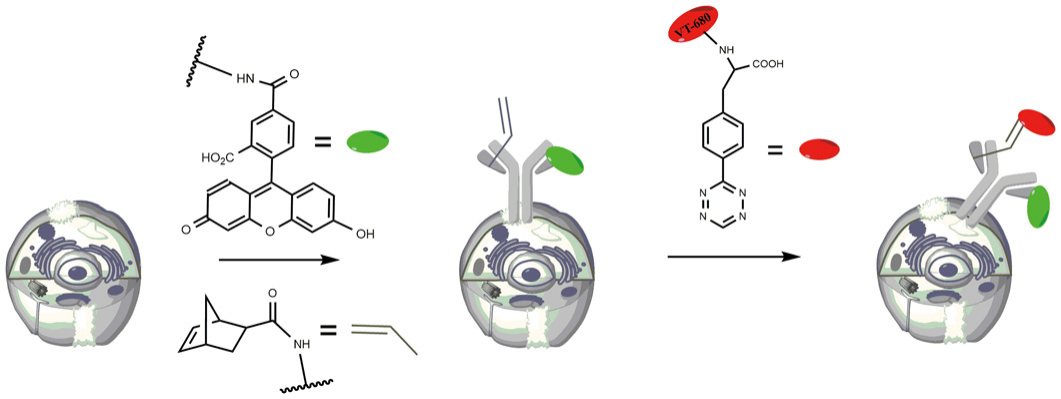
The compound 2 reacted with norbornene to label cancer cells. Cancer cells A549, which overexpressed EGFR, were exposed to the Cetuximab antibodies modified with norbornene and 5-carboxyfluorescein (green). In the next step, the pretargeted cells were labeled with a tetrazine bearing a fluorophore such as VT680 (red).

Specific labeling of living cells was one of the most promising applications in bioorthogonal chemistry [31]. The tetrazine-containing amino acid exhibited excellent aqueous solubility (10–20 mg/ml at 25°C) and was more suitable for cell experiment in physiological conditions compared with 3-(p-benzylamino)-1, 2, 4, 5-tetrazine. To demonstrate the application of the tetrazine-containing amino acid in living cells marking, we chose to label A549 lung cancer cells, which overexpressed epidermal growth factor receptors (EGFR), with the specific anti-EGFR monoclonal antibody cetuximab. Cetuximab was marked with norbornene derivative and 5-carboxyfluorescein N-succinimidyl ester. To label cells pretargeted with norbornene-bearing antibodies, the tetrazine-containing amino acid **2** was conjugated to a commercially available far-red indocyanine fluorophore, Vivo-Tag 680 (Fig 2). When A549 cells were incubated in solution with modified cetuximab and subsequently labeled with tetrazine-VT680, we can observe cellular changes by fluorescence microscopy in rhodamine channel and near infrared (NIR) channel, respectively. Control groups with either unlabeled cetuximab and tetrazine-VT680 or norbornene-cetuximab and unlabeled VT680 were also performed.

**Fig 3:**
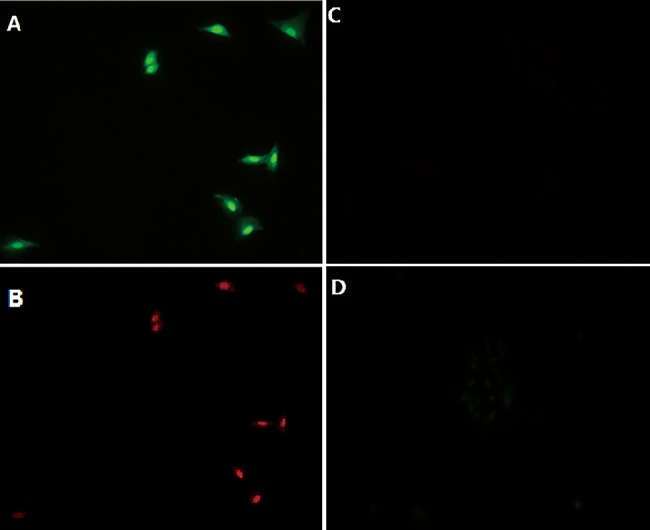
Fluorescent microscope images of A549 lung cancer cells after pretargeting Cetuximab antibodies modified with 5-carboxyfluorescein and norbornene and subsequent labeling with tetrazine-VT680. (A) Rhodamine channel. (B) Near-IR channel (tetrazine-VT680). Images of control experiments were also taken in near infrared channel. (C) Control experiment with unlabeled cetuximab and tetrazine-VT680. (D) Control experiment with norbornene-cetuximab and unlabeled VT680.

Results showed that the antibody could be visualized clearly in rhodamine channel and covalently binding of tetrazine-VT680 could be monitored apparently in the NIR channel. While the controls showed no NIR channels (Fig 3). These experiments demonstrated the specificity and sensitivity of the reaction between tetrazine-containing amino acid and norbornene-modified antibody in the complex biological systems of living cells. Consequently, we concluded that this reaction of tetrazine-containing amino acid was suitable for the in vitro experiments, and might also provide an effective way to label cells in the complex intracellular environment.

### Synthesis and stability of protected tetrazine-containing amino-acids 5 and 6 (S4 File)

To test whether tetrazine-containing amino acid could be used for selective peptide modification, we investigated the stability of tetrazine in acidic or alkaline conditions, a prerequisite in solid-phase peptide synthesis. In the synthesis process, there were an extensive range of resins designed to generate peptide acids or peptide amides via an Fmoc or Boc synthetic strategy. We first synthesized Fmoc-protected tetrazine-containing amino acid **5** and Boc-protected tetrazine-containing amino acid **6** to investigate whether tetrazine can stably exist in 20% piperidine/DMF or 50% TFA/DCM for deprotection of Fmoc or Boc group. Following with the exponential decay of the tetrazine absorbance at 523 nm at room temperature, we observed that the tetrazine of compound **5** decomposed almost 100% within 60 seconds in the presence of 20% piperidine/DMF at room temperature (**Fig M in S4 File**). However, the tetrazine of compound **6** showed excellent stability in 50% TFA/DCM with little decomposition after prolonged exposure at room temperature (**Fig N in S4 File**). Moreover, the tetrazine of compound **6** showed fairly stability in DIEA/DMF (**Fig O in S4 File**), an environment used for the formation of amide bond in solid phase peptide synthesis.

### Synthesis and the in vitro effects on the small intestines of peptides 7 and 8 (S5 File)

We focused on the design and syntheses of the tetrazine-peptide conjugates next. In solid phase peptide synthesis, Sieber resin was selected as the biological macromolecular material, since peptide can be cleaved easily from Sieber resin under acidic conditions [32]. Substance P (SP) was found by Euler in the brain and intestine tissue, and the active SP influenced intestinal smooth muscle contraction and vasodilatation and lower blood pressure, and so on [33–35]. C-terminal pentapeptide of SP (5Phe-4Phe-3Gly-2Leu-1Met amide), the core sequence, still retained some features of SP [36], so the pentapeptide was selected as the target peptide for tetrazine coupling. Eventually we obtained corresponding tetrazine-containing C-terminal pentapeptide using Boc-protected tetrazine-containing amino acid **6** instead of the C-terminal pentapeptide of SP on solid phase peptide synthesis.

**Fig 4:**
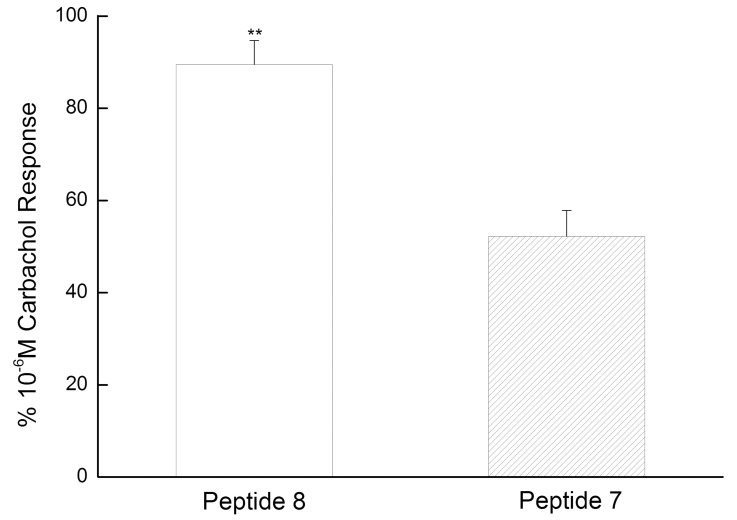
Peptides 7 and 8 (both with 10 μM) for small intestine contraction with acetylcholine (10 μM) as positive control.

To investigate whether the tetrazine-containing peptide **8** was able to elicit activity at comparable effect to that of the parent pentapeptide, we obtained the parent peptide **7** according to the procedures described above. Peptides **7** and **8** were used, respectively, to perform a small intestine contraction experiment with acetylcholine as positive control. The experiments indicated that the enhancement of small intestine contraction caused by tetrazine-containing peptide **8** (10 μM, 89.50 ± 5.20%) was greater than parent peptide **7** (10 μM, 52.22 ± 5.64%) (Fig 4).

## Conclusion

In conclusion, we synthesized the compound (S)-3-(4-(1, 2, 4, 5-tetrazin-3-yl) phenyl)-2-aminopropanoic acid in the catalysis of elemental sulfur with L-4-cyano-phenylalanine, formamidine acetate and anhydrous hydrazine. Importantly, results implied that no racemization occurred in the synthesis process. Tetrazine-containing amino acid possessed excellent stability in biological media and acidic solution, which laid the foundation for cancer cells labeling and peptide modification. Moreover, we also proved that the tetrazine-modified peptide **8** showed more significant biological activity than the parent peptide **7**, suggesting that tetrazine-containing amino acid might be useful in the modification of peptides and proteins in the future work.

## Supporting Information

S1 File Synthesis and enantiomeric purity analysis of tetrazine-containing amino acid 2. ESI-MS spectrum (**1**) and Analytical HPLC spectrum (**2**) of compound **2 (Fig A).** ^1^H NMR spectrum (**1**) and ^13^C NMR spectrum (**2**) of compound **2 (Fig B).** Analytical HPLC spectrum (**1**) and UV-vis absorption spectrum (**2**) of peak 1 and peak 2 **(Fig C).** Analytical HPLC spectrum (**1**) and UV-vis absorption spectrum (**2**) of FDAA **(Fig D)**. ESI-MS spectrum of peak 1 (**1**) and peak 2 (**2**) **(Fig E)**.
(DOCX)

S2 File Stability and kinetic experiments of tetraine-containing amino acid 2. UV-vis absorption spectrum of compound **2 (Fig F).** The stability of **2** in PBS at 20.0 ± 0.1°C monitored at 523 nm **(Fig G)**. Kinetics of the reaction of **2** (0.4 mM) and **3** (4 mM) in deionized water, monitored by UV-vis at 523 nm **(Fig H)**. ESI-MS spectrum of compound **4** and its’ isomers **(Fig I).**
(DOCX)

S3 File Tetrazine-containing amino acid 2 in the lung cancer cell labeling. (DOCX)

S4 File Synthesis and stability of protected tetrazine-containing amino-acids 5 and 6. ESI-MS spectrum of compound **5** (**1**) and compound **6** (**2**) **(Fig J).** ^1^H NMR spectrum (**1**) and ^13^C NMR spectrum (**2**) of compound **5 (Fig K).** ^1^H NMR spectrum (**1**) and ^13^C NMR spectrum (**2**) of compound **6 (Fig L).** The stability of **5** in 20% piperidine / DMF at 20.0 ± 0.1°C monitored at 523 nm **(Fig M).** The stability of **6** in 50% TFA/DCM at 20.0 ± 0.1°C monitored at 523 nm **(Fig N).** The stability of **6** in DIEA/DMF at 20.0 ± 0.1°C monitored at 523 nm **(Fig O).**
(DOCX)

S5 File Synthesis and the in vitro effects on the small intestines of peptides 7 and 8. Analytical HPLC spectrum of peptides **7** (**1**) and **8** (**2**) **(Fig P).** ESI-MS spectrum of peptides **7** (**1**) and **8** (**2**) **(Fig Q).**
(DOCX)
