# Supplementary material for: Tetrazine-Containing Amino Acid for Peptide Modification and Live Cell Labeling
Source: PLoS One. 2015 Nov 4;10(11):e0141918. doi: 10.1371/journal.pone.0141918 (PMC4633098; doi:10.1371/journal.pone.0141918)
Supplement: S1 File — ESI-MS spectrum (1) and Analytical HPLC spectrum (2) of compound 2 (Fig A). 1H NMR spectrum (1) and 13C NMR spectrum (2) of compound 2 (Fig B). Analytical HPLC spectrum (1) and UV-vis absorption spectrum (2) of peak 1 and peak 2 (Fig C). Analytical HPLC spectrum (1) and UV-vis absorption spectrum (2) of FDAA (Fig D). ESI-MS spectrum of peak 1 (1) and peak 2 (2) (Fig E). (DOCX) [file pone.0141918.s001.docx]

**Synthesis of tetrazine-containing amino acid 2**

Compound **2** was prepared according to our previous work in the synthesis of tetrazine derivatives [1].

Compound **1** (1.43 g, 7.5 mmol), formamidine acetate (3.12 g, 30 mmol) and sulfur (240 mg, 7.5 mmol) were mixed in a round-bottom flask under the protection of nitrogen. Anhydrous hydrazine (9.42 ml, 300 mmol) was added to the flask, and the mixture became yellow viscous gradually with stirring for 22 h at room temperature. We added 45 ml acetic acid to dissolve the yellow viscous material, and then obtained the supernatant solution after centrifugal settling. Mixed sodium nitrite (2.59 g, 37.5 mmol) in 4.5 ml water fully, and dropped it into the above supernatant solution slowly under the condition of an ice-water bath, and then generated a purple solution after 30 minutes’ reaction. Evaporated organic solvent out in vacuum condition as far as possible. Added ethanol (300 ml) to the reaction mixture, filtered the solid off and washed thoroughly with ethanol (100 ml). And finally, 1.19 g purple powder (pure product **2,** Figure A) was gained, which can be used in the next step without further purification. Yield 64.9 %. R_f_ 0.54 (N-butyl alcohol: glacial acetic acid: water = 4: 1: 1).

ESI-MS (m/z, Figure A 1): calcd. for C_11_H_11_N_5_O_2_ 245.09; found [M + H]^+^: 246.1.

^1^H NMR (DMSO, 295 K, 300 MHz, δ Figure B 1): 10.62 (s, 1H), 8.47 (d, J = 8.1 Hz, 5H), 7.59 (d, J = 8.1 Hz, 2H), 4.32(s, 1H), 3.25 (d, 2H).

^13^C NMR (100 MHz, DMSO-d^6^, Figure B 2): δ 170.26, 165.36, 158.12, 140.26, 130.76, 130.59, 127.92, 52.87, and 35.74.

**Enantiomeric purity of tetrazine-containing amino acid 2**

Derivatization of an amino acid with FDAA produced a diastereomer referred to as DNPA-amino acid. Tetrazine-containing amino acid (1.9 mg, 7.8 μM) was placed in a 2 ml plastic tube. 300 μl acetone solution of FDAA (3 mg, 11 μM) was added to the tube, with the molar ratio of FDAA to amino acid 1.4: 1, followed with NaHCO_3_ (1 M, 60 μl, 60 μM). The contents were mixed at 30 – 40 °C for one hour with intensive mixing. After cooling to room temperature, HCl (2 M, 30 μl, 60 μM) was added to the reaction mixture. The DNPA-amino acid can be separated and estimated by HPLC with a linear gradient of MeCN from 5 to 95 % in 20 min at a flow rate of 1 ml/min and UV detection at 340 nm. The results showed two peaks of absorption spectra in RP-HPLC, and the two peaks were measured with mass spectrometry to confirm the purity. The results proved that the product was found only in peak 2, but not in peak 1 (Figure C). It has been recognized that the peak 2 was the FDAA derivative of tetrazine-containing amino acid. Moreover, acetone solution of FDAA was injected for HPLC as well, and the results showed that the FDAA had the same absorption spectra (Figure D) and retention behavior as that with peak 1. It is evident that the tetrazine-containing amino acid was L-isomer and might be used for peptide synthesis. Analytical RP-HPLC (Figure C 1): R_t_ 16.70 min.

ESI-MS (m/z, Figure E): calcd. for C_20_H_19_N_9_O_7_ 497.14; found [M + H]^+^: 498.14.

**Reference**

1. Sauer J, Heldmann DK, Hetzenegger J, Krauthan J, Sichert H, Schuster J. 1, 2, 4, 5-Tetrazine: Synthesis and Reactivity in [4 + 2] Cycloadditions. Eur J Org Chem. 1998; 1998(12):2885-96.

**1**

**
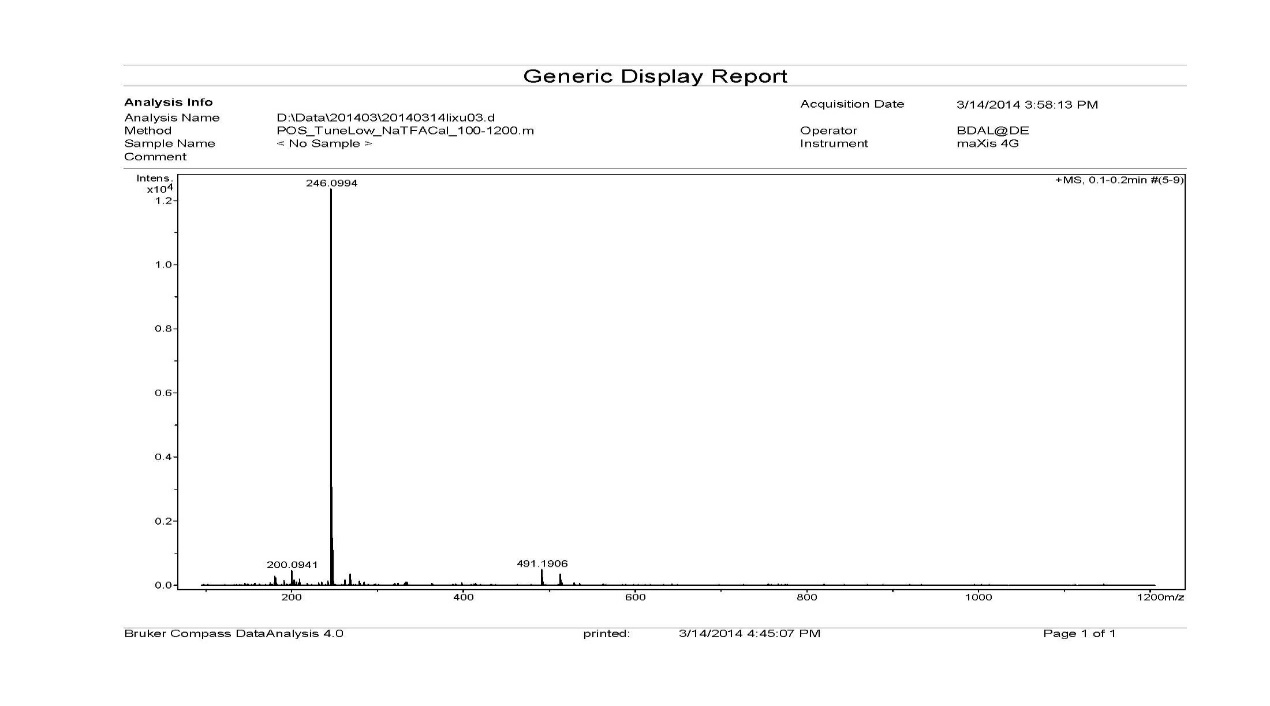
**

**2**


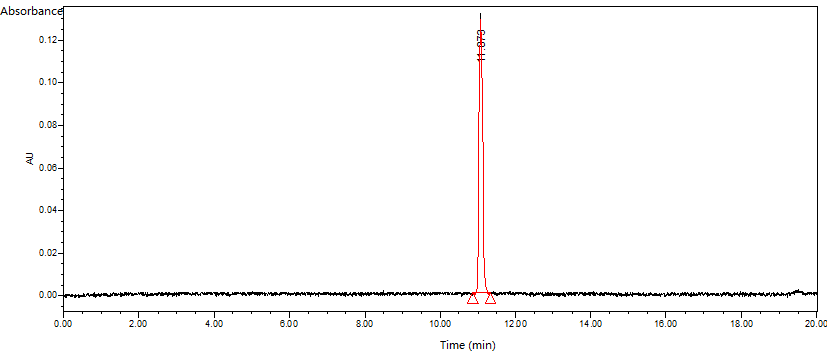


**Figure A.** ESI-MS spectrum (**1**) and Analytical HPLC spectrum (**2**) of compound **2**

**1**

**
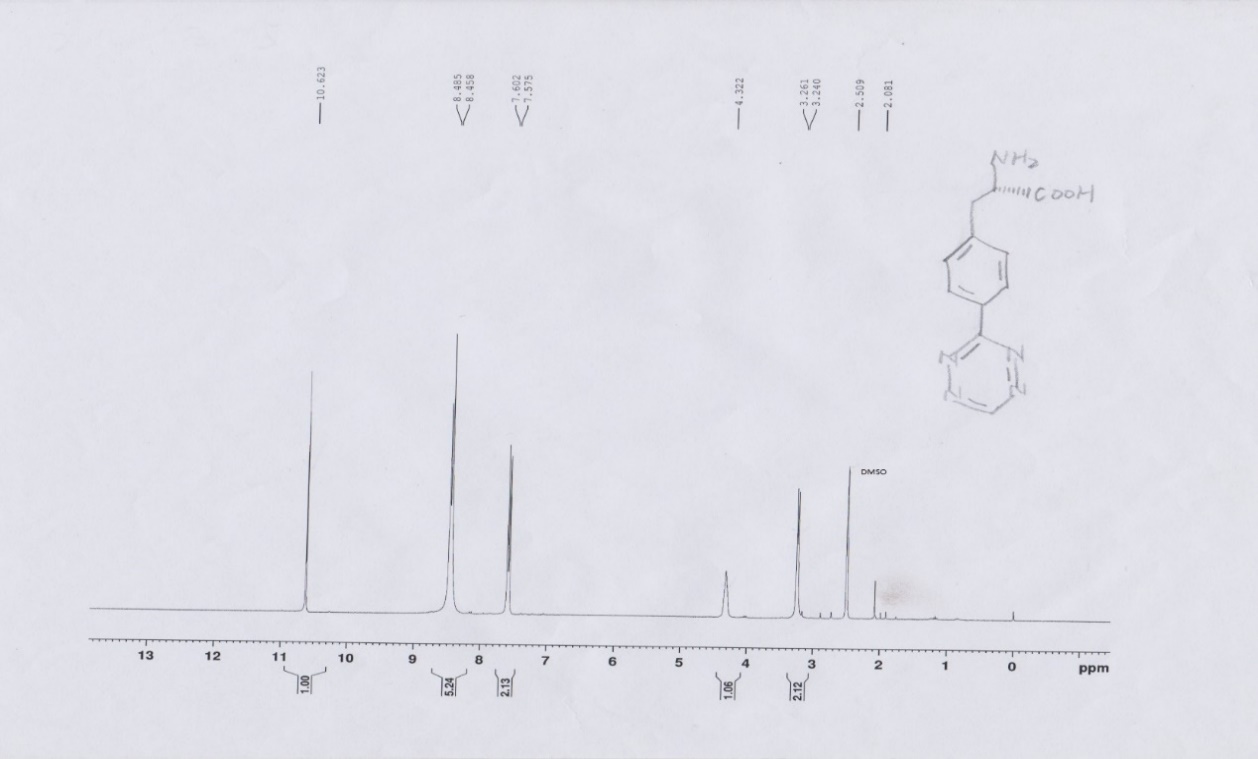
**

**2**

**
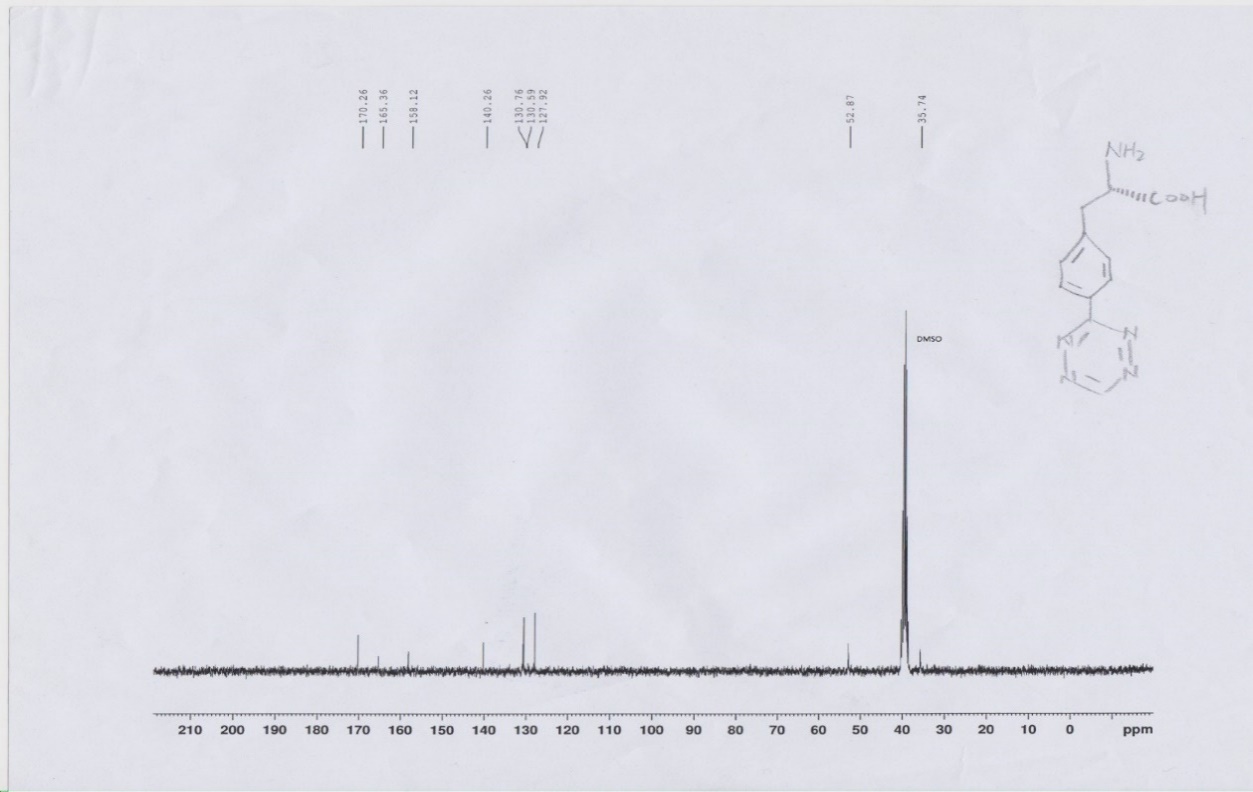
**

**Figure B.** ^1^H NMR spectrum (**1**) and ^13^C NMR spectrum (**2**) of compound **2**

**1**

**
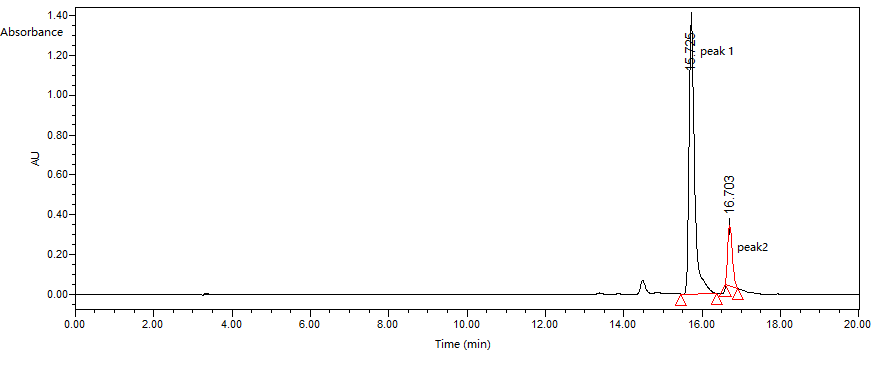
**

**2**

**
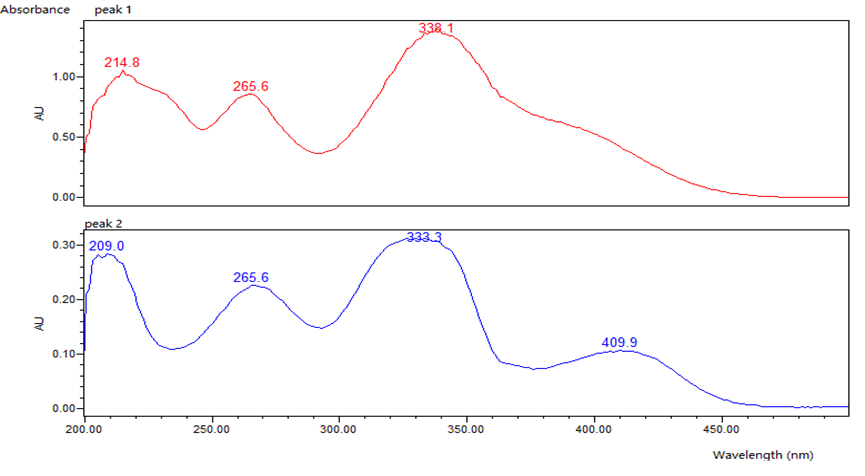
**

**Figure C.** Analytical HPLC spectrum (**1**) and UV-vis absorption spectrum (**2**) of peak 1 and peak 2

**1**

**
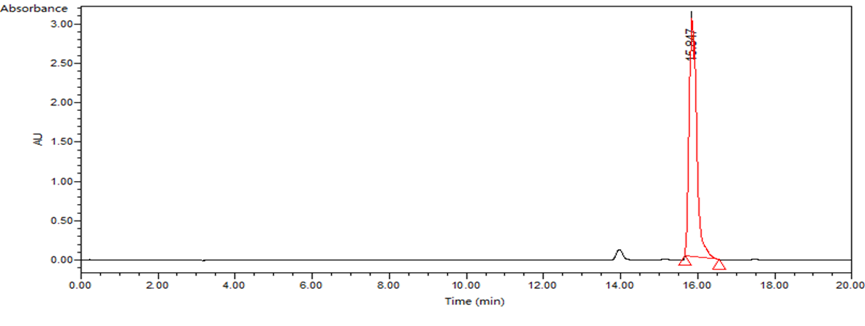
**

**2**

**
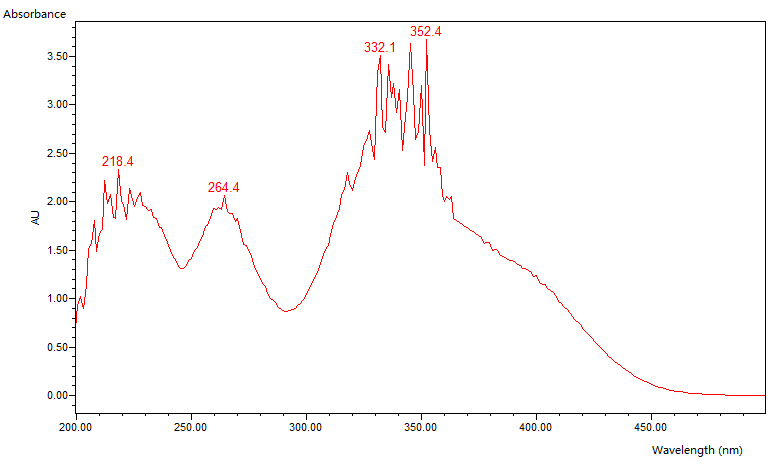
**

**Figure D.** Analytical HPLC spectrum (**1**) and UV-vis absorption spectrum (**2**) of FDAA

**1**

**
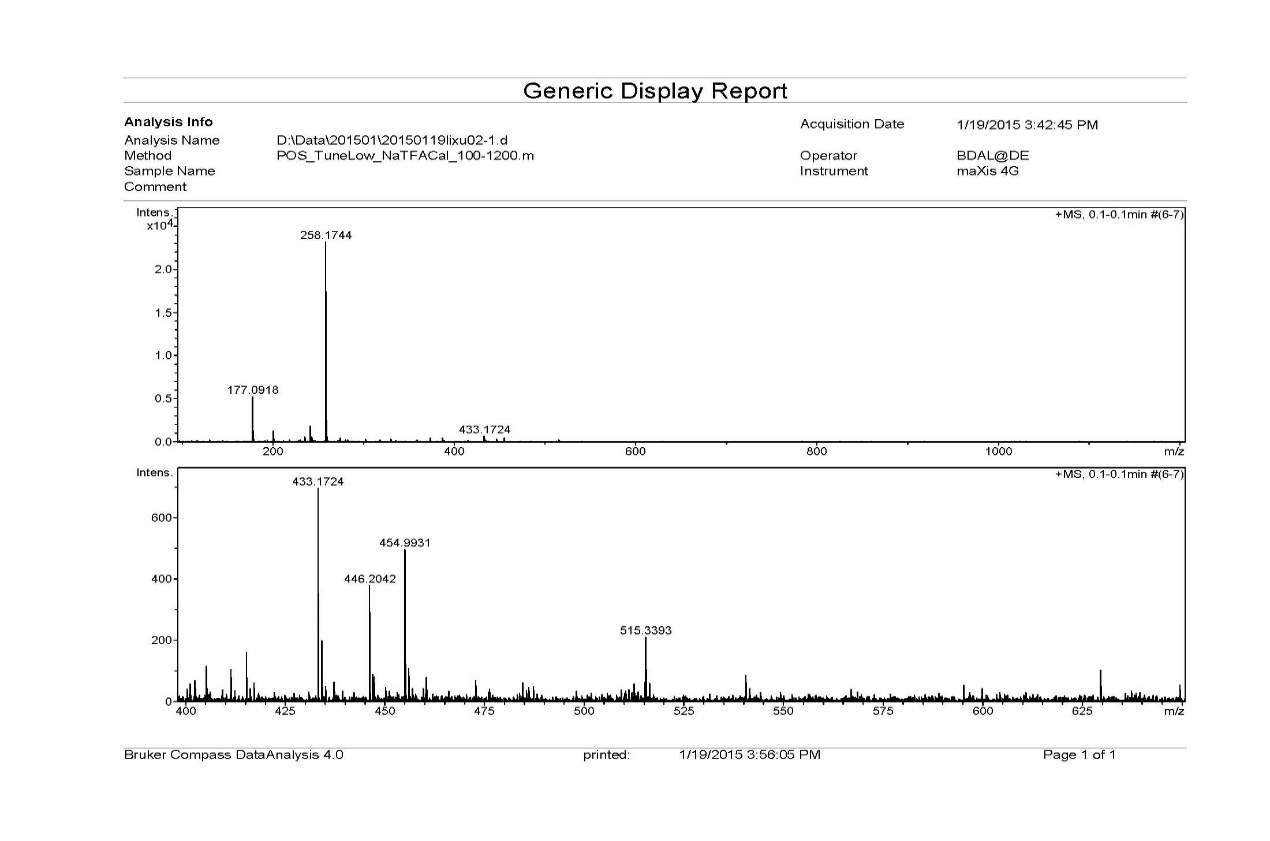
**

**2**

**
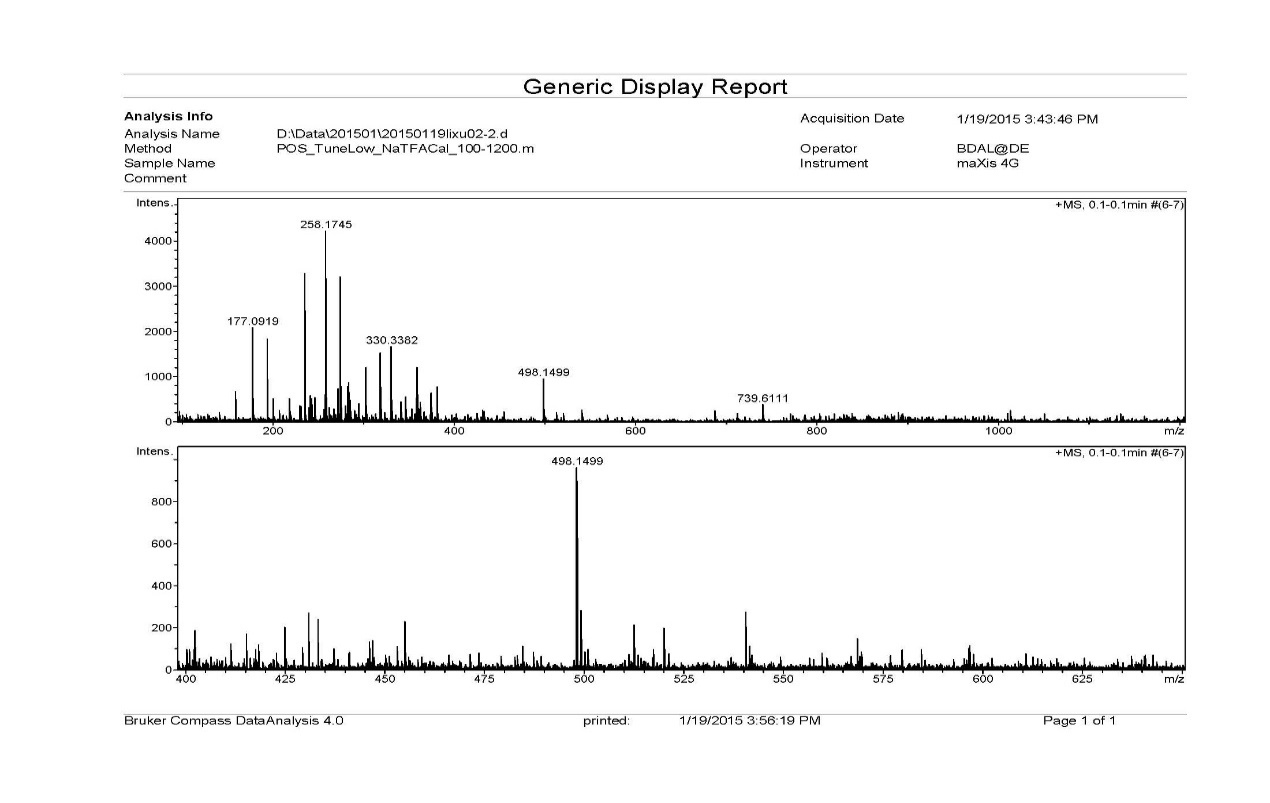
**

**Figure E.** ESI-MS spectrum of peak 1 (**1**) and peak 2 (**2**)
