# Supplementary material for: Tetrazine-Containing Amino Acid for Peptide Modification and Live Cell Labeling
Source: PLoS One. 2015 Nov 4;10(11):e0141918. doi: 10.1371/journal.pone.0141918 (PMC4633098; doi:10.1371/journal.pone.0141918)
Supplement: S2 File — UV-vis absorption spectrum of compound 2 (Fig F). The stability of 2 in PBS at 20.0 ± 0.1°C monitored at 523 nm (Fig G). Kinetics of the reaction of 2 (0.4 mM) and 3 (4 mM) in deionized water, monitored by UV-vis at 523 nm (Fig H). ESI-MS spectrum of compound 4 and its’ isomers (Fig I). (DOCX) [file pone.0141918.s002.docx]

**Stability of** **tetrazine-containing amino acid 2**

The stabilities of selected tetrazines in PBS were measured with Agilent 8453 UV-vis spectrophotometer (Figure F). The compounds were added to PBS at 37 °C in a capped quartz cuvette with the final concentration of 1 mmol. The decrease of the tetrazine absorbance measured at 523 nm was monitored for 6 h at 37 °C (Figure G). Three independent trials (containing three wells for each tetrazine) were conducted and the average of the three wells was calculated for each trial. The relative percent tetrazine remaining can be seen Figure G, where the error was the standard deviation of the three trials.

**Kinetic Experiments of the reaction between 2 and 3**

All kinetic experiments were conducted under pseudo-first order conditions. The pseudo first order rate constants were obtained by plotting the natural log of the concentration of the limiting reactant versus time in seconds. The second-order rate constants and half-lives were extrapolated from the rate measurements derived under pseudo-first order conditions.

As illustrated in Fig. 1, the isopyknic PBS of **2** (0.4 mmol) and **3** (4 mmol) were mixed into the cuvette at room temperature. The absorption spectrum of the starting material **2** under the absorption peak at 523 nm, because the product **4** and its isomers had no absorption at 523nm. The absorbance value was recorded every 20 seconds to monitor the change of absorbance spectrum as the reaction progress. An exponential curve was used for calculating the reaction rate, the calculated pseudo first order reaction constant *k*_obs_ = 2.678 × 10^-3^ ± 6 × 10^-6^ _M_^-1^⋅_S_^-1^, the half-life t_1/2_ = 260 s, and further calculations obtained second-order reaction rate constant *k*_2_ = 1.339 ± 0.003 _M_^-1^⋅_S_^-1^ (Figure H).

ESI-MS (m/z, Figure I): calcd. for C_19_H_21_N_3_O_4_ 355.15; found [M + H]^+^: 356.16, [2M + H]^+^: 711.31, [3M + H]^+^: 1066.44.

**
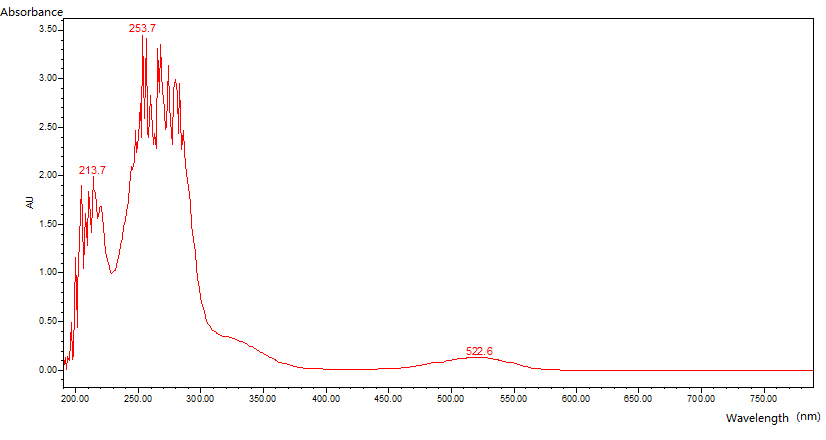
**

**Figure F.** UV-vis absorption spectrum of compound **2**

**
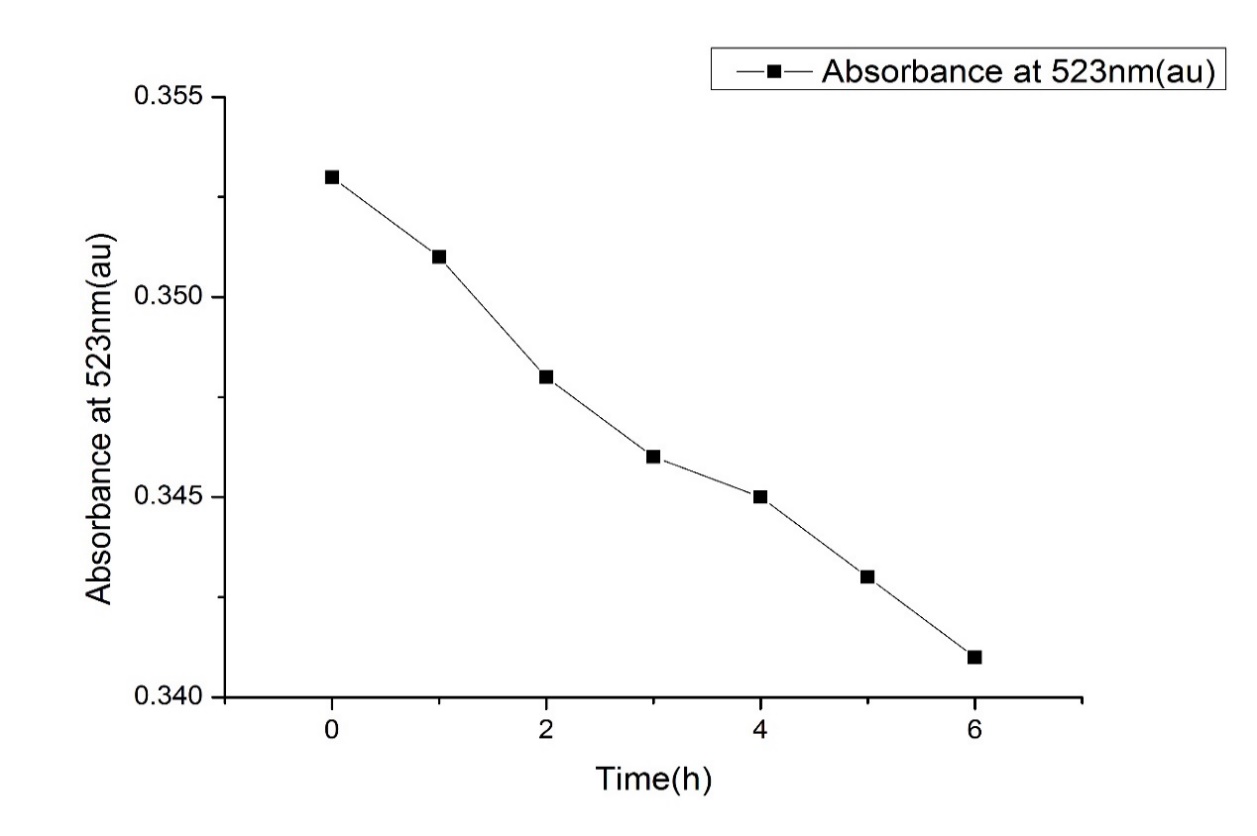
**

**Figure G.** The stability of **2** in PBS at 20.0 ± 0.1 °C monitored at 523 nm. After 6 h, a 3.3 % decrease in absorption was observed.
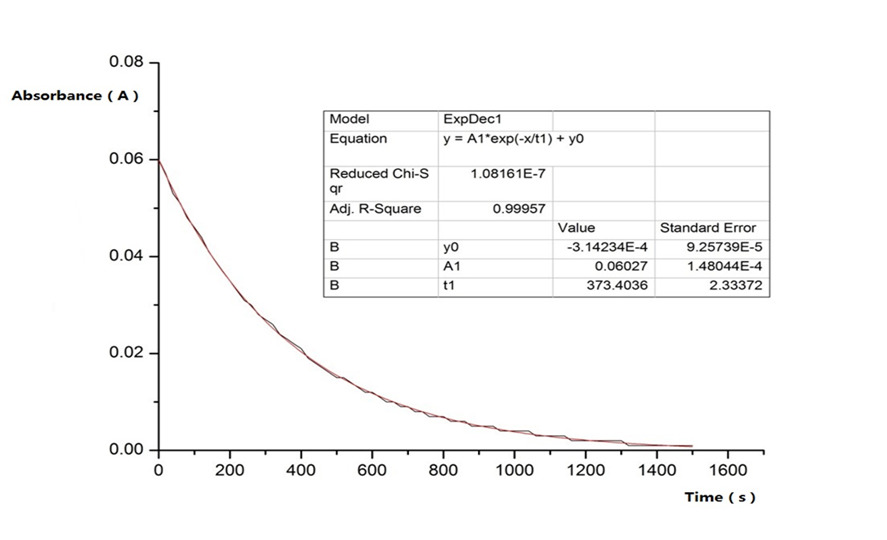


**Figure H.** Kinetics of the reaction of **2** (0.4 mM) and **3** (4 mM) in deionized water, monitored by UV-vis at 523 nm. Kinetic runs were performed in triplicate (all data was shown above) and fitted to an observed first-order rate constant (kobs), which was used to calculate the second order rate constant.

**
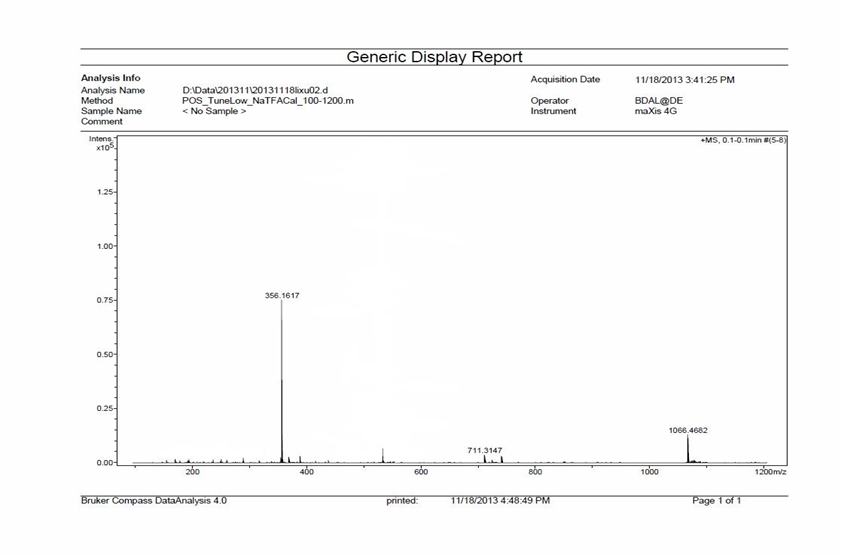
**

**Figure I.** ESI-MS spectrum of compound **4** and its’ isomers
