# Supplementary material for: Tetrazine-Containing Amino Acid for Peptide Modification and Live Cell Labeling
Source: PLoS One. 2015 Nov 4;10(11):e0141918. doi: 10.1371/journal.pone.0141918 (PMC4633098; doi:10.1371/journal.pone.0141918)
Supplement: S3 File — (DOCX) [file pone.0141918.s003.docx]

**Tetrazine-containing amino acid 2 in the lung cancer cell** **labeling**

The synthesis of L-4-tetrazine-Phe-VT680 and norbornene-cetuximab were performed as previously reported [1, 2].

VT680 NHS (1 mg) reacted with **2** (1.58 mg) in PBS solution (250 μl, 0.1 M), and finally we obtained the product L-4-tetrazine-Phe-VT680. N, N'-disuccinimidyl carbonate and **3** were reacted in acetonitrile with pyridine as the base to give the norbornene succinimidyl ester. Added 5(6)-carboxyfluorescein succinimidyl ester and norbornene succinimidyl ester in a flask, to react with the amino group of the antibody cetuximab to give a modified cetuximab which connected with 5(6)-carboxyfluorescein and norbornene. A549 cells and modified antibody were incubated at 37 °C and washed to clean the unbounded antibody of the cell surface 3 hours later. L-4-tetrazine-Phe-VT680 reacted with norbornene group of the modified antibody in 10 % fetal bovine serum and Hanks' balanced salt solution at 37 °C for 1 hour via IED-DA reaction (Fig. 2). Then a washing step was performed to remove unreacted L-4-tetrazine-Phe-VT680. Either unlabeled cetuximab and tetrazine-VT680 or norbornene-cetuximab and unlabeled VT680 was taken as the control. In the end, cellular changes were monitored by fluorescence microscopy in rhodamine channel and near infrared channel (NIR), respectively. The results showed that the antibody could be visualized clearly in rhodamine channel and covalently bound tetrazine-VT680 could be monitored apparently in NIR channel, while control experiments with no NIR fluorescence (Fig. 3).

**Reference**

1. Devaraj NK, Weissleder R, Hilderbrand SA. Tetrazine-based cycloadditions: application to pretargeted live cell imaging. Bioconjug Chem. 2008;19(12):2297-9.

2. Devaraj NK, Upadhyay R, Haun JB, Hilderbrand SA, Weissleder R. Fast and sensitive pretargeted labeling of cancer cells through a tetrazine/trans-cyclooctene cycloaddition. Angew Chem Int Ed Engl. 2009;48(38):7013-6.
