# Supplementary material for: Tetrazine-Containing Amino Acid for Peptide Modification and Live Cell Labeling
Source: PLoS One. 2015 Nov 4;10(11):e0141918. doi: 10.1371/journal.pone.0141918 (PMC4633098; doi:10.1371/journal.pone.0141918)
Supplement: S4 File — ESI-MS spectrum of compound 5 (1) and compound 6 (2) (Fig J). 1H NMR spectrum (1) and 13C NMR spectrum (2) of compound 5 (Fig K). 1H NMR spectrum (1) and 13C NMR spectrum (2) of compound 6 (Fig L). The stability of 5 in 20% piperidine / DMF at 20.0 ± 0.1°C monitored at 523 nm (Fig M). The stability of 6 in 50% TFA/DCM at 20.0 ± 0.1°C monitored at 523 nm (Fig N). The stability of 6 in DIEA/DMF at 20.0 ± 0.1°C monitored at 523 nm (Fig O). (DOCX) [file pone.0141918.s004.docx]

**Synthesis of protected tetrazine-containing amino-acid 5**

The compound **2** (490.4 mg, 2 mmol) was suspended in a 1: 1 mixture of water and 1, 4-dioxane (50 ml) in a round bottom flask. Sodium bicarbonate (420 mg, 5 mmol) was added in the condition of an ice-water bath, followed by Fmoc-OSu (1.02 g, 3 mmol). The mixture was stirred vigorously and the reaction was completed in 6 to 10 hours at ambient temperature in a variety of solvents. Rotary evaporation to remove as much of 1, 4-dioxane, and washed the mixture twice with ether. Hydrochloric acid (1 mol/L) was added slowly to the solution under an ice-water bath to adjust pH to 3. Then ethyl acetate was added to extract the purple product. The product was washed twice with saturated aqueous sodium chloride, and then dried over anhydrous sodium sulfate overnight. After the rotary evaporation and chromatography purification of the excess ethyl acetate, we obtained the product 602.3 mg with yield 64.5 %. R_f_ 0.49 (petroleum ether: ethyl acetate: acetic acid = 20: 20: 1).

ESI-MS (m/z, Figure J 1): calcd. for C_26_H_21_N_5_O_4_ 467.16; found [M + Na]^+^: 490.1.

^1^H NMR (DMSO, 295K, 300 MHz, δ, Figure K 1): 10.58 (s, 1H), 8.42 (d, J = 8.1 Hz, 2H), 7.88-7.82 (m, 3H), 7.66-7.57 (m, 4H), 7.40-7.26 (m, 4H), 4.34-4.14 (m, 5H), 3.39-2.99 (m, 2H), 2.51 (s, 1H).

^13^C NMR (100 MHz, DMSO-d^6^, Figure K 2): δ 173.14, 165.39, 158.02, 155.90, 143.68, 143.50, 140.61, 130.26, 129.96, 127.59, 127.54, 126.97, 125.19, 120.03, 65.53, 55.18, 46.49, 36.43.

**Synthesis of protected tetrazine-containing amino-acid 6**

To a stirred ice-cold solution of compound **2** (490.4 mg, 2 mmol) in a 1: 1 mixture of water and dioxane (50 ml), Sodium bicarbonate (420 mg, 5 mmol) was added followed by Boc_2_O (873 mg, 4 mmol, 2.0 eq) in dioxane (5 ml) in portions within 5 min. The mixture was stirred for 8 h under nitrogen gas at room temperature. Rotary evaporation to remove as much of 1, 4-dioxane, and washed the mixture twice with ether. Hydrochloric acid (1 mol/L) was added slowly to the solution under an ice-water bath to adjust pH to 3, then added ethyl acetate to extract the purple product. Washed the product twice with saturated aqueous sodium chloride, and then dried over anhydrous sodium sulfate overnight. Excess of ethyl acetate was removed by rotary evaporation and purified by chromatography on a silica gel column. Finally, we got the product 498.5 mg with yield 72.3 %. R_f_ 0.45 (petroleum ether: ethyl acetate: acetic acid = 20: 20: 1).

ESI-MS (m/z, Figure J 2): calcd. for C_16_H_19_N_5_O_4_ 345.14; found [M + Na]^+^: 368.13, [2M + Na]^+^: 713.27, [3M+Na]^+^: 1035.42

^1^H NMR (DMSO, 295K, 300 MHz, δ, Figure L 1): 10.58 (s, 1H), 8.42 (d, J = 8.1 Hz, 2H), 7.56 (d, J = 8.1 Hz, 2H), 7.24 (d, J = 8.4 Hz, 1H), 4.24-4.16 (m, 1H), 3.20-3.14 (m, 1H), 3.01-2.93 (m, 1H), 2.51 (s, 1H), 1.32 (s, 9H).

^13^C NMR (100 MHz, DMSO-d^6^，Figure L 2): δ 173.35, 165.42, 158.03, 155.41, 143.51, 130.22, 129.94, 127.56, 78.06, 54.80, 38.36, 28.07.

**Stability of** **protected tetrazine-containing amino-acid 5 and 6**

The stability of tetrazine-containing amino acid **5** and **6** in different solutions (20 % piperidine/DMF, 50 % TFA/DCM and DIEA/DMF for Figure M – O, respectively) were all measured with the same method described in S2 File.

**1**

**
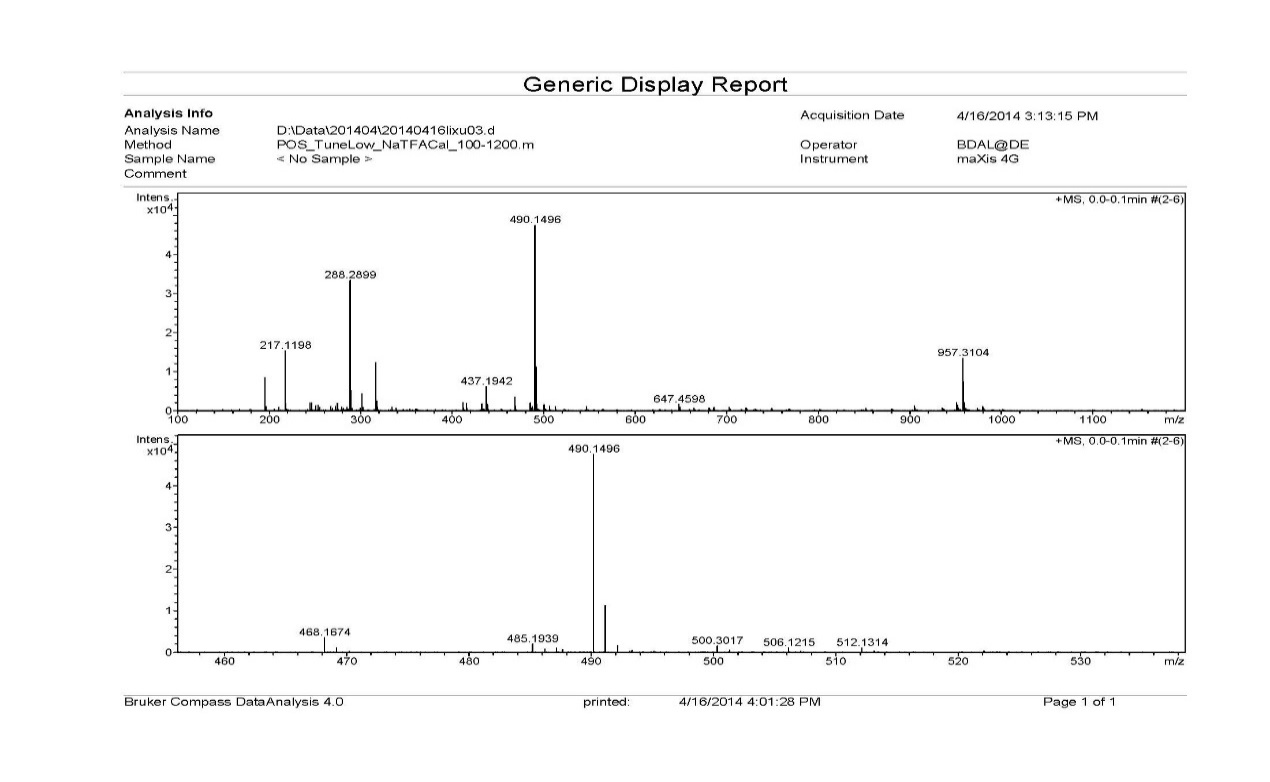
**

**2**

**
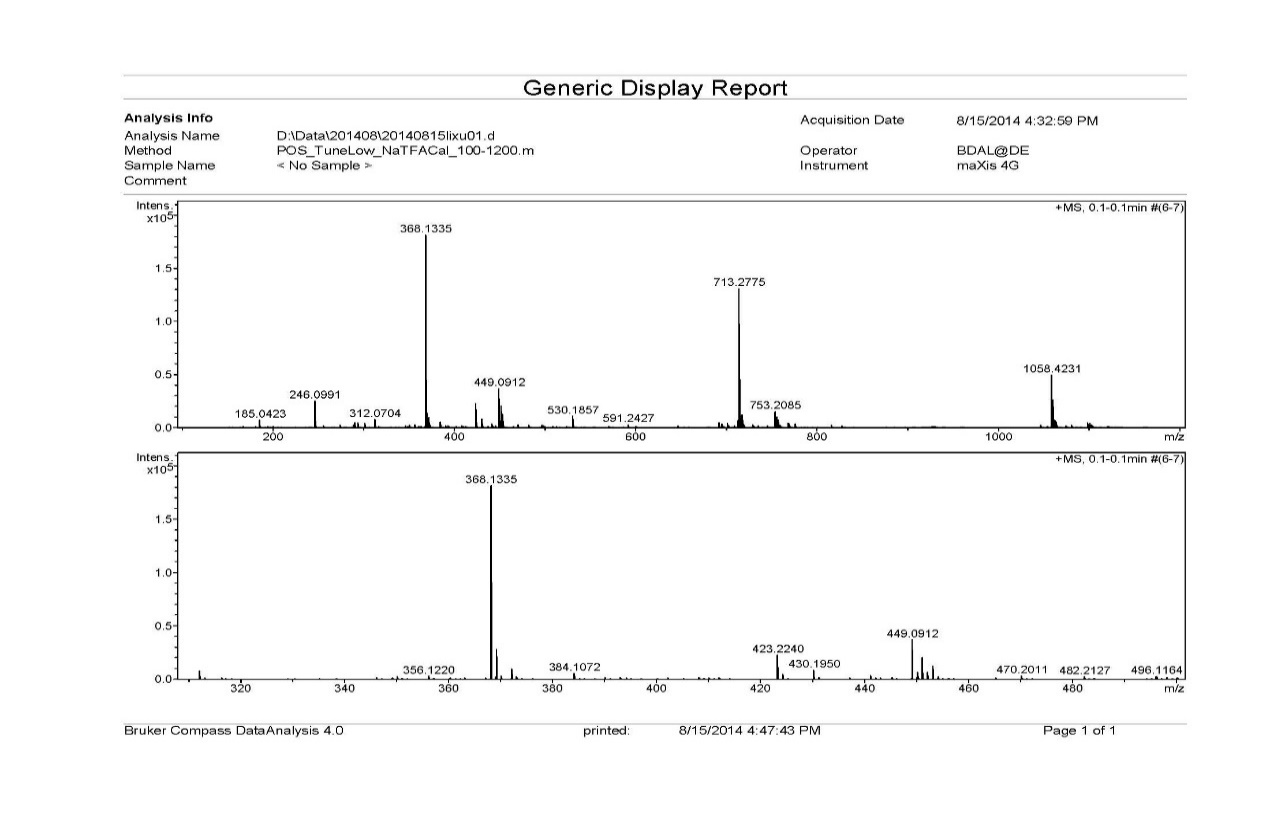
**

**Figure J**. ESI-MS spectrum of compound **5** (**1**) and compound **6** (**2**) **1**

**
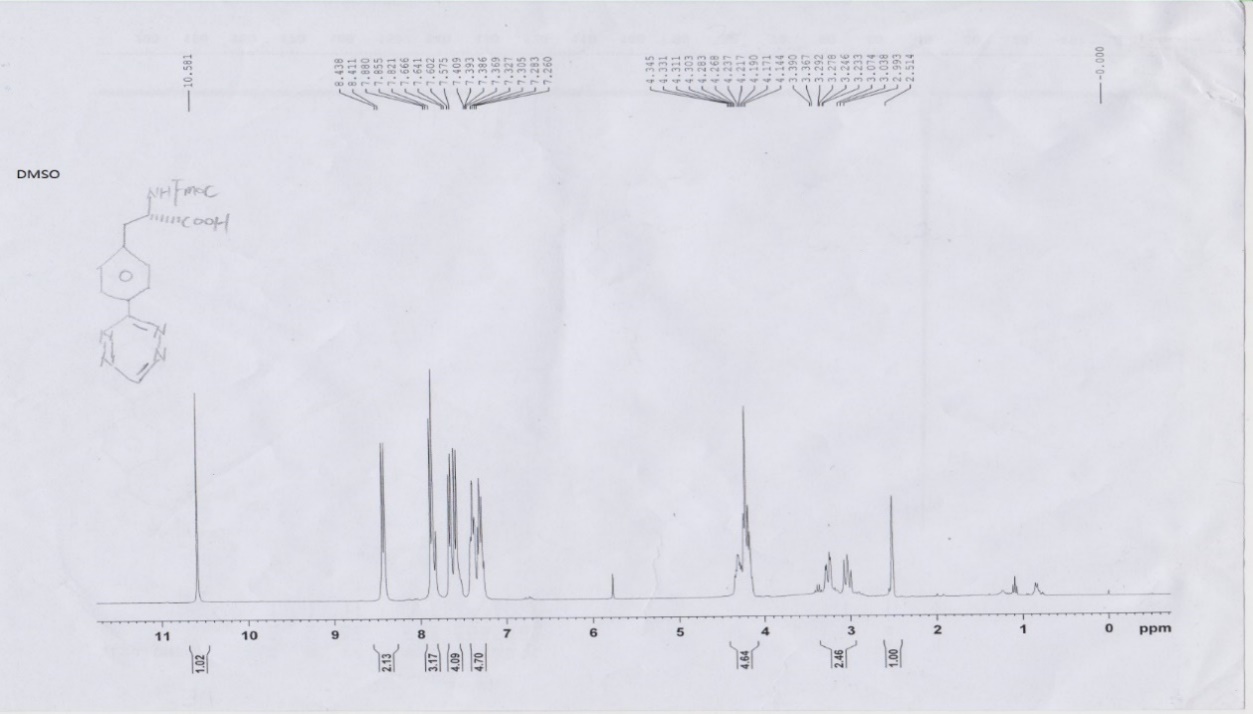
**

**2**

**
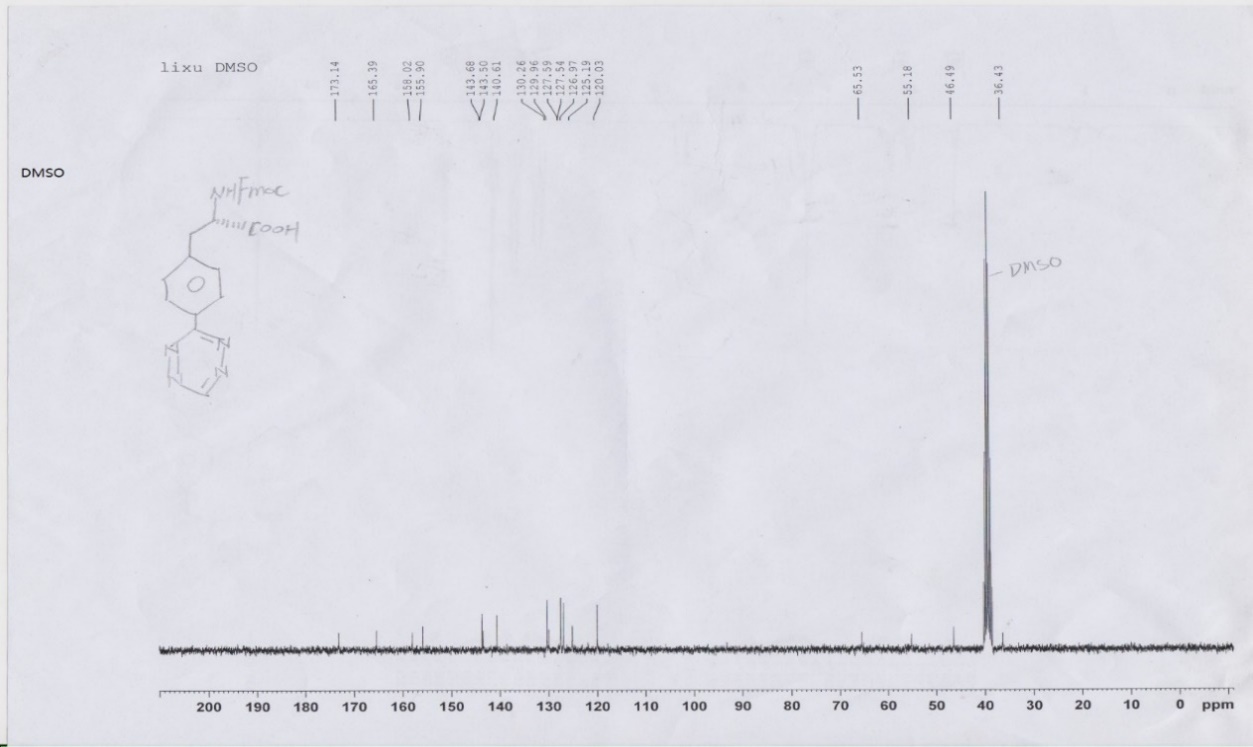
**

**Figure K.** ^1^H NMR spectrum (**1**) and ^13^C NMR spectrum (**2**) of compound **5**

**1**

**
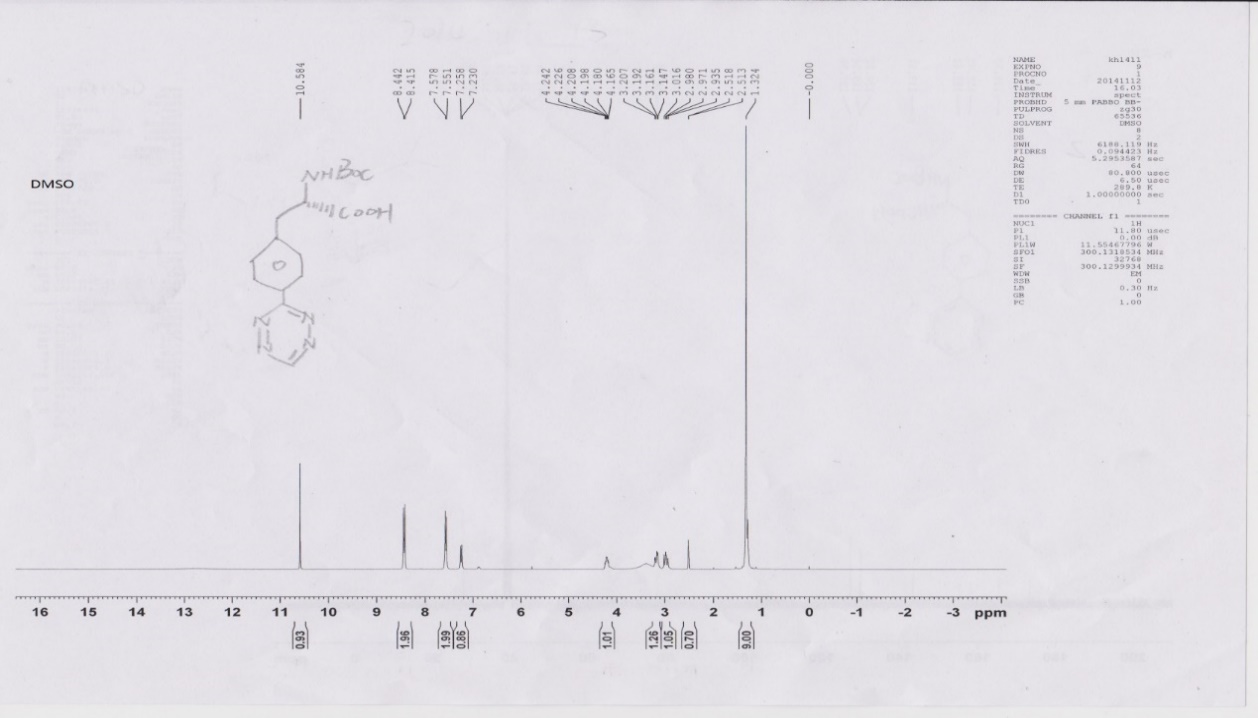
**

**2**


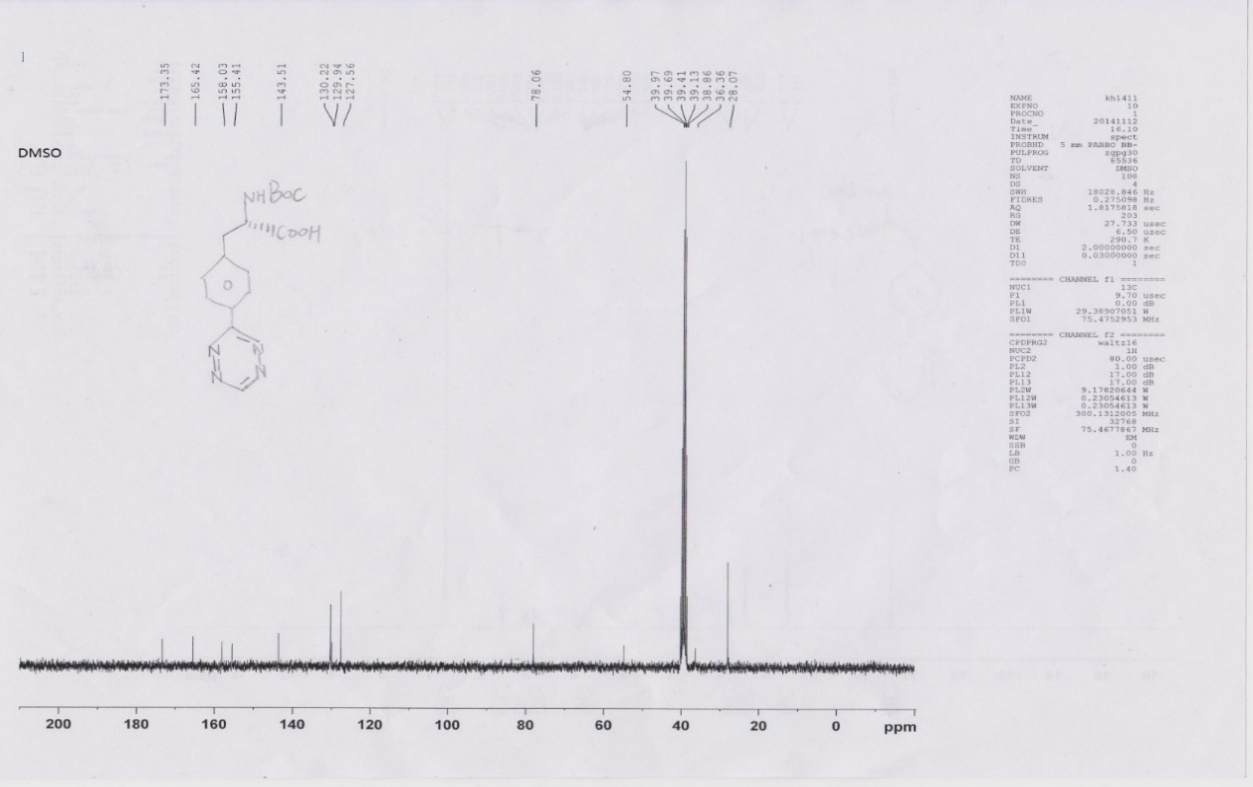


**Figure L.** ^1^H NMR spectrum (**1**) and ^13^C NMR spectrum (**2**) of compound **6**

**
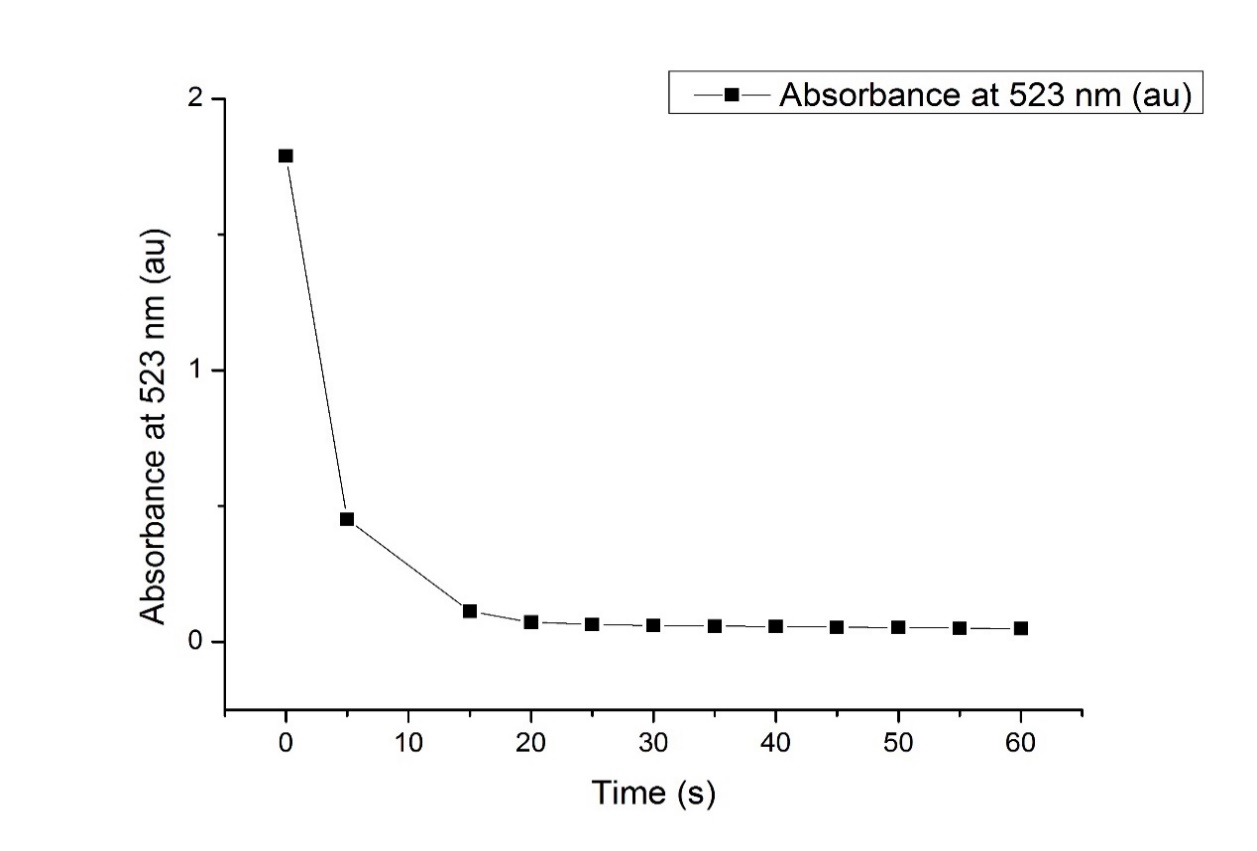
**

**Figure M.** The stability of **5** in 20 % piperidine / DMF at 20.0 ± 0.1 °C monitored at 523 nm. After 60 S, almost a 100 % decrease in absorption was observed.

**
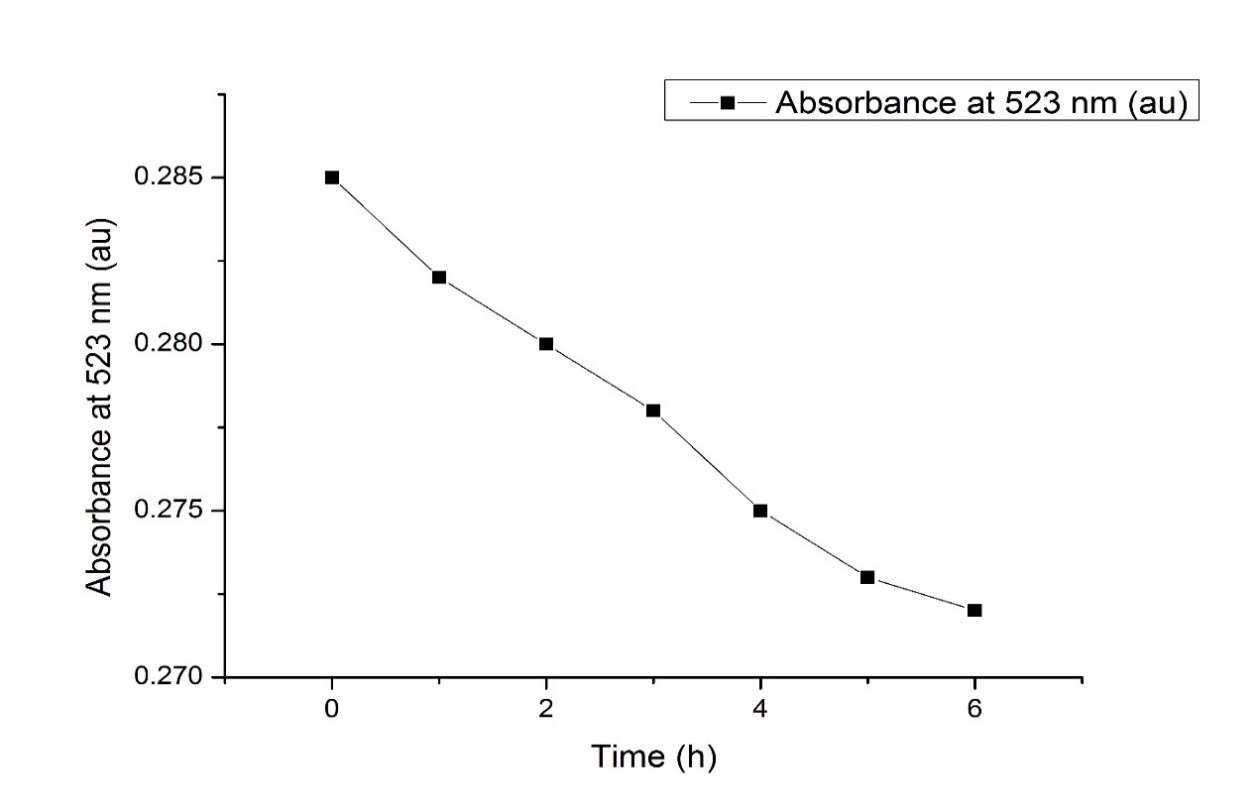
**

**Figure N.** The stability of **6** in 50 % TFA/DCM at 20.0 ± 0.1 °C monitored at 523 nm. After 6 h, a 4.5 % decrease in absorption was observed.

**
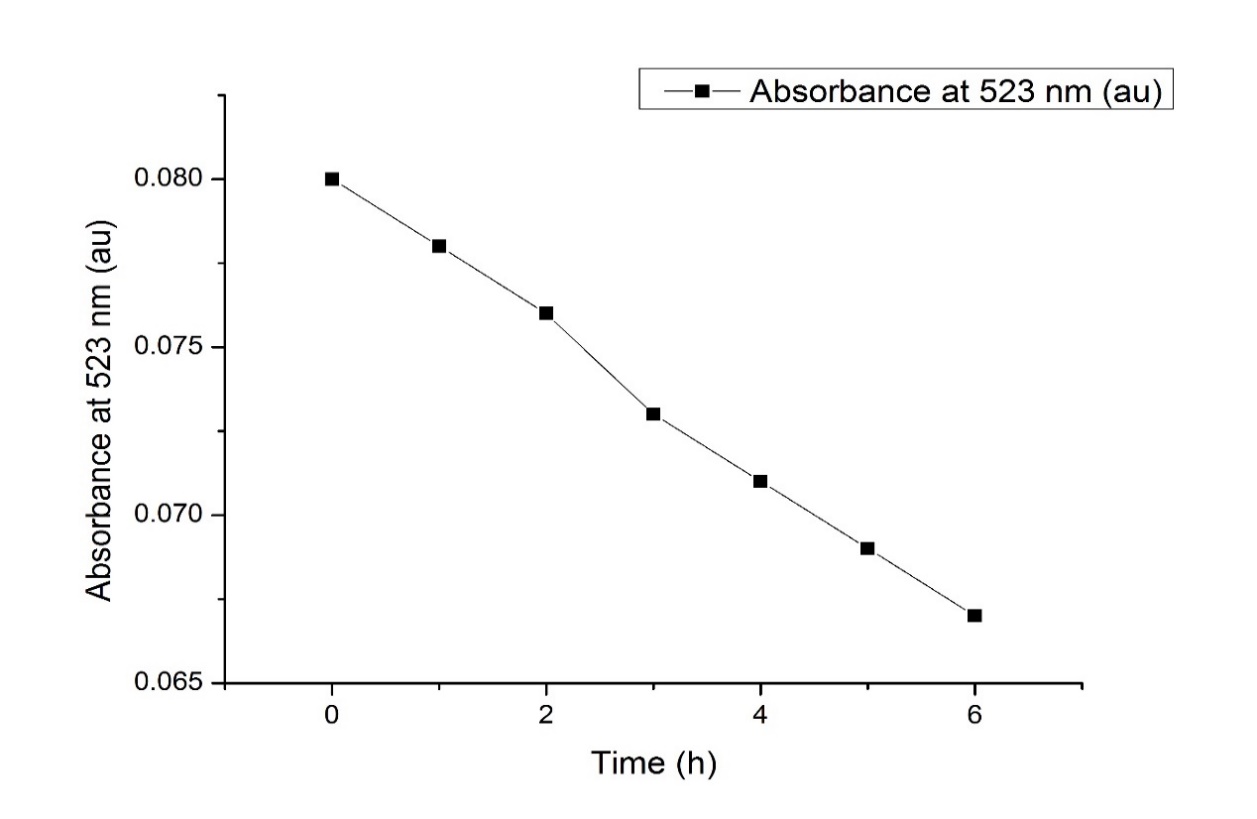
**

**Figure O.** The stability of **6** in DIEA/DMF at 20.0 ± 0.1 °C monitored at 523 nm. After 6 h, a 16 % decrease in absorption was observed.
