# Supplementary material for: Tetrazine-Containing Amino Acid for Peptide Modification and Live Cell Labeling
Source: PLoS One. 2015 Nov 4;10(11):e0141918. doi: 10.1371/journal.pone.0141918 (PMC4633098; doi:10.1371/journal.pone.0141918)
Supplement: S5 File — Analytical HPLC spectrum of peptides 7 (1) and 8 (2) (Fig P). ESI-MS spectrum of peptides 7 (1) and 8 (2) (Fig Q). (DOCX) [file pone.0141918.s005.docx]

**Synthesis of tetrazine-containing peptide 8**

Added Sieber Amide Resin (SD = 0.5 mmol/g, 0.03 mmol) and appropriate dichloromethane (DCM) to a 5 ml BD syringe with the sieve, then shaking the mixture with 640 r/min for 2 hours to swell the resin. Drained the solvent, then the reactor was added with 5 ml 20 % piperidine of N,N'-dimethyl formamide (DMF) solution (V/V). After 20 minutes’ stir at room temperature, we used the Kaiser test to examine whether the Fmoc group was completely removed. Fmoc-Met-OH, Fmoc-Leu-OH, Fmoc-Gly-OH and Fmoc-Phe-OH were added to the reaction container gradually. Fmoc amino acids (2 eq) were activated for 10 min with DIC (2 eq) and HOBt (2 eq) in DMF and added the amino-functionalized resin in DMF. The reaction mixture was shook for 2 h and washed with DMF (3 × 10 ml) and DCM (2 × 10 ml). The Fmoc protecting groups were removed with 20 % piperidine in DMF. Each coupling reaction or Fmoc deprotection was also monitored by the Kaiser test. After the synthesis of tetrapeptide, compound **6** (0.06 mmol, 1.5 eq) was added to the syringe. The reaction was stirred vigorously and completed in an hour at ambient temperature. Cleavage of peptide from the resin and deprotection of Boc groups were performed by treatment with TFA/DCM (50: 50) for 1.5 h, then precipitated with cold Et_2_O. The precipitation was collected by centrifugation and suspended in cold Et_2_O. This procedure was repeated twice and the crude product was purified by preparative RP-HPLC under the conditions described above. Yield: 14.1 mg, 68.8 %; analytical RP-HPLC (Figure P 2): 97.4 % R_t_ 14.34 min;

ESI-MS (m/z, Figure Q 2 ): calcd. for C_33_H_44_N_10_O_5_S 692.32, found [M + H]^+^: 693.33.

**Synthesis of parent peptide 7**

With the similar method of tetrazine-containing peptide **8**, we have also synthesized parent peptide (^5^Phe-^4^Phe-^3^Gly-^2^Leu-^1^Met amide) on a sieber resin (SD = 0.5 mmol/g, 0.05 mmol). Yield: 28.2 mg 92.1 %; analytical RP-HPLC (Figure P 1): 96.2 % R_t_ 14.16 min; ESI-MS (m/z, Figure Q 1): calcd. for C_31_H_44_N_6_O_5_S 612.31, found [M + H]^+^: 613.31.

**Peptides 7 and 8 for the** **small intestine contraction**

The in vitro experiments were performed following with the previous methods [1]. Briefly, adult mice were sacrificed by cervical dislocation. Segments of small intestine of mice (approximately 1 cm in length) were obtained from ileum, flushed of their contents and trimmed of mesentery. The preparations were suspended under 1 g tension with silk thread in 5 ml organ baths containing Kreb’s solution (NaCl, 118 mM; KCl, 4.74 mM; CaCl_2_, 2.54 mM; KH_2_PO_4_, 1.19 mM; MgSO_4_, 1.20 mM; NaHCO_3_, 25 mM; glucose, 11 mM) maintained at 37 °C and bubbled with 95 % O_2_/5 % CO_2_. One end of the preparation was attached to the bottom of the organ bath, while the other end was connected to a strain gauge transducer for the recording of isometric tension. The samples were allowed to equilibrate for 60 min before the drugs with a wash-out at 15 min intervals.

The preparation obtained from ileum was contracted with parent peptide **7** (10 μM) and the amplitude of the contraction was recorded with 10 μM acetylcholine as the positive control. Parent peptide **7** was washed out of the organ bath and tension returned to baseline. Tetrazine-containing peptide **8** for the small intestine contraction was measured with the same method. The results were expressed as means ± SEM. The experiment revealed that both peptides **7** (10 μM) and **8** (10 μM) can enhance the small intestine contraction. In addition, the contraction enhancement induced by peptide **8** (89.50 ± 5.20 %) was far greater than the parent peptide **7** (52.22 ± 5.64 %). Difference was considered to be significant at *P* < 0.05 (Fig. 4).

**Reference**

1. Yu Y, Cui Y, Wang X, Lai LH, Wang CL, Fan YZ, et al. In vitro characterization of the effects of endomorphin 1 and 2, endogenous ligands for mu-opioid receptors, on mouse colonic motility. Biochemical pharmacology. 2007;73(9):1384-93. doi: 10.1016/j.bcp.2007.01.011. PubMed PMID: 17274956.

**1**

**
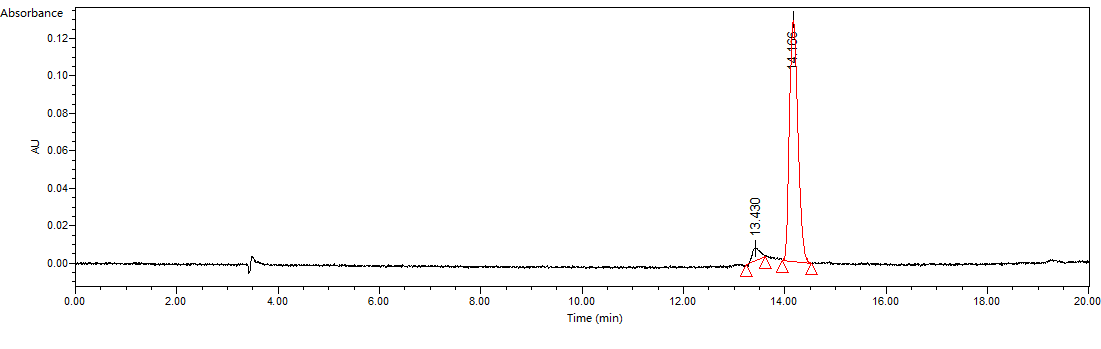
**

**2**

**
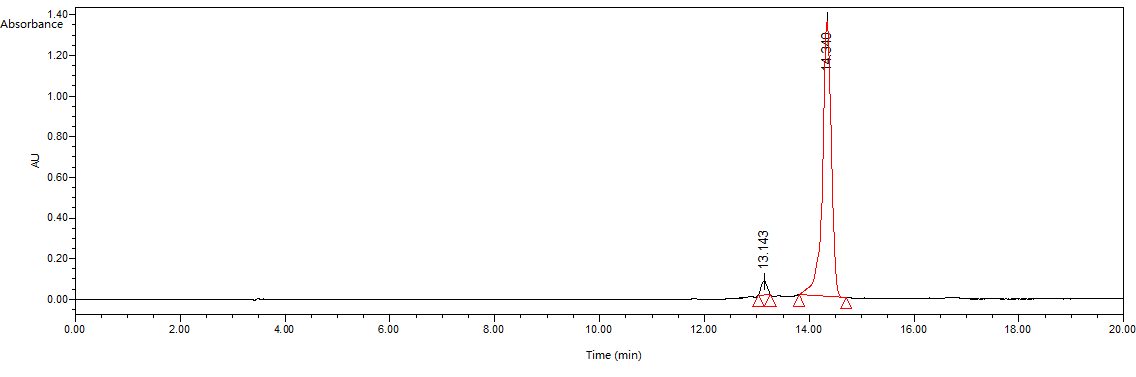
**

**Figure P.** Analytical HPLC spectrum of peptides **7** (**1**) and **8** (**2**) **1**

**
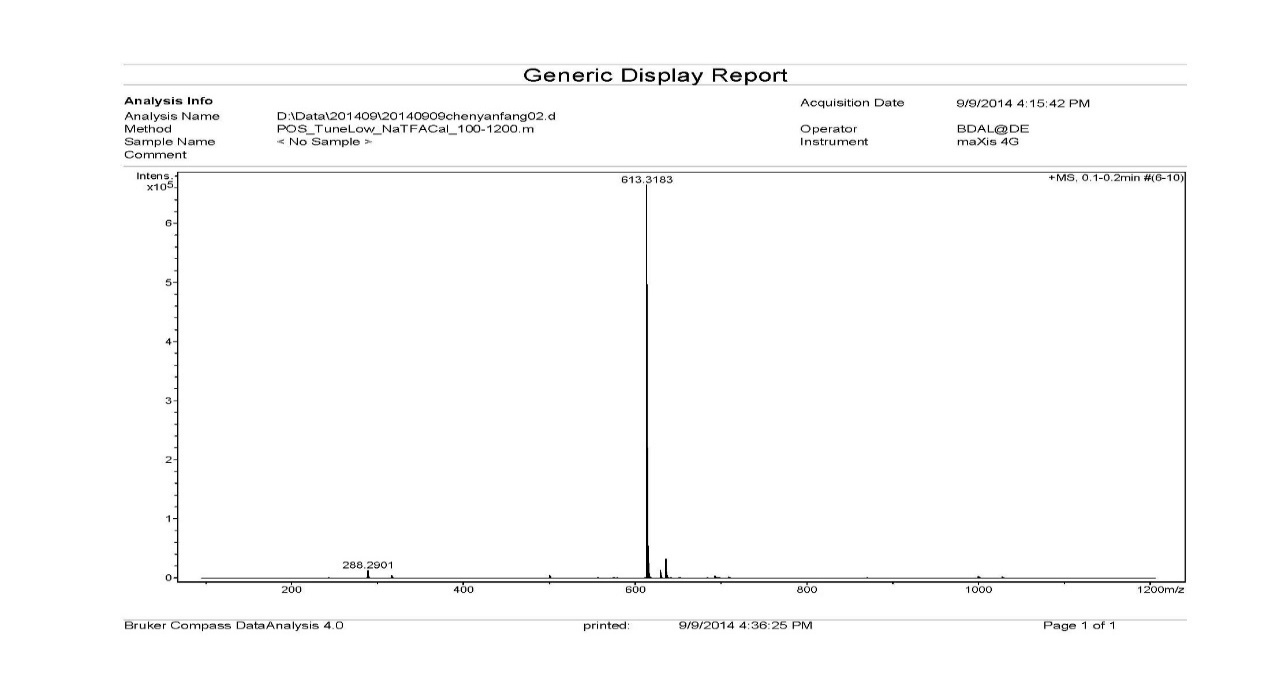
**

**2**

**
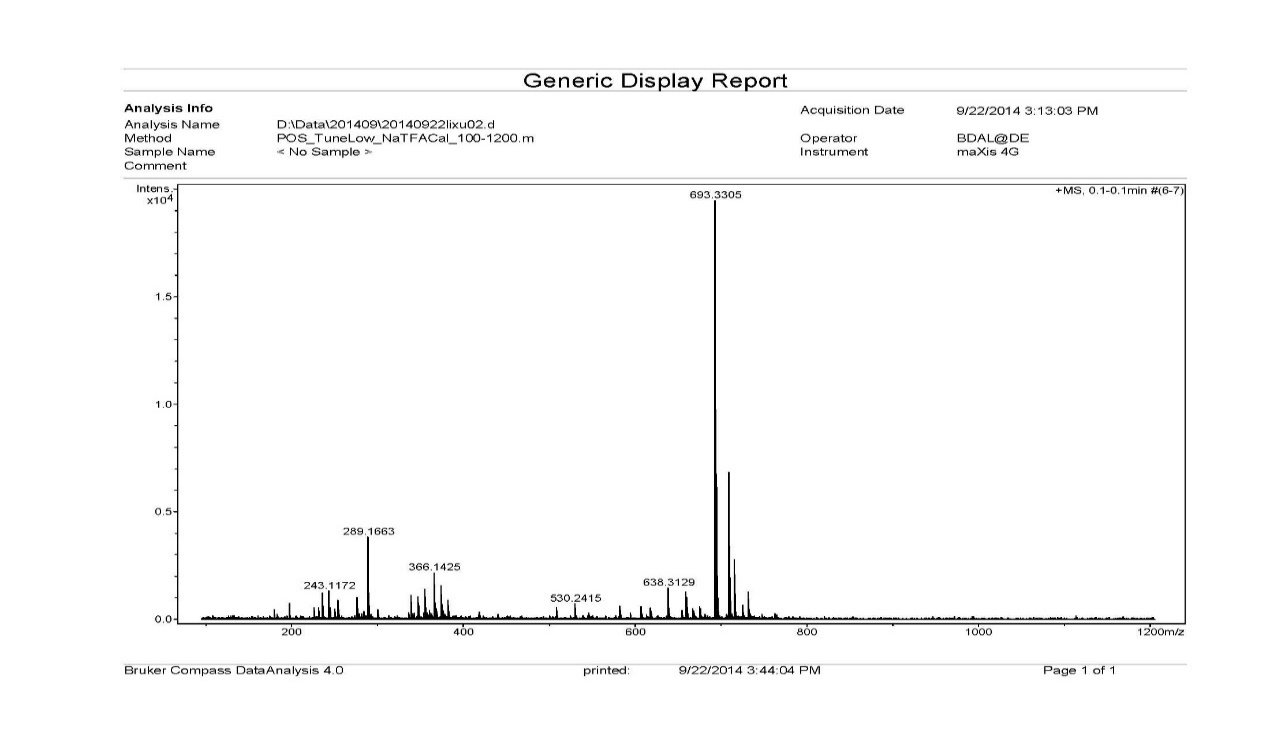
**

**Figure Q.** ESI-MS spectrum of peptides **7** (**1**) and **8** (**2**)
